# Combating Actions of Green 2D-Materials on Gram Positive and Negative Bacteria and Enveloped Viruses

**DOI:** 10.3389/fbioe.2020.569967

**Published:** 2020-09-28

**Authors:** Manjot Singh, Carla Zannella, Veronica Folliero, Rocco Di Girolamo, Francesco Bajardi, Annalisa Chianese, Lucia Altucci, Achille Damasco, Maria Rosaria Del Sorbo, Concetta Imperatore, Manuela Rossi, Mohammadhassan Valadan, Michela Varra, Alessandro Vergara, Guanluigi Franci, Massimiliano Galdiero, Carlo Altucci

**Affiliations:** ^1^Laboratory of Bio-Nano-Photonics, Department of Physics “Ettore Pancini”, University of Naples “Federico II”, Naples, Italy; ^2^Department of Experimental Medicine, University of Campania “Luigi Vanvitelli”, Naples, Italy; ^3^Department of Chemical Sciences, University of Naples “Federico II”, Naples, Italy; ^4^Istituto Nazionale di Fisica Nucleare, Naples, Italy; ^5^Department of Precision Medicine, University of Campania “Luigi Vanvitelli”, Naples, Italy; ^6^Istituto Statale d’Istruzione Superiore “Leonardo da Vinci”, Naples, Italy; ^7^Department of Pharmacy, University of Naples “Federico II”, Naples, Italy; ^8^Department of Earth Science, Environment and Resources, University of Naples “Federico II”, Naples, Italy; ^9^Department of Medicine, Surgery and Dentistry “Scuola Medica Salernitana”, University of Salerno, Baronissi, Italy

**Keywords:** 2D materials, biocompatibility, antibacterial, antivirals, DLVO, *E. coli*, *S. aureus*, HSV-1 virus

## Abstract

Interactions of novel bi-dimensional nanomaterials and live matter such as bacteria and viruses represent an extremely hot topic due to the unique properties of the innovative nanomaterials, capable in some cases to exhibit bactericide and antiviral actions. The interactions between bacteria and viruses and two dimensional nanosheets are here investigated. We extensively studied the interaction between a gram-negative bacterium, *Escherichia coli*, and a gram-positive bacterium, *Staphylococcus aureus*, with two different types of 2D nanoflakes such as MoS_2_, belonging to the Transition Metal Dichalcogenides family, and Graphene Oxide. The same two types of nanomaterials were employed to study their antiviral action toward the Herpes simplex virus type-1, (HSV-1). The experimental results showed different bactericide impacts as well as different antiviral power between the two nanomaterials. The experimental findings were interpreted in bacteria on the base of the Derjaguin–Landau–Verwey–Overbeek theory. A simple kinetic model of bacterial growth in the presence of the interacting nanosheets is also elaborated, to explain the observed results. The experimental results in viruses are really novel and somewhat surprising, evidencing a stronger antiviral action of Graphene Oxide as compared to MoS_2_. Results in viruses are complicated to quantitatively interpret due to the complexity of the system under study, constituted by virus/host cell and nanoflake, and due to the lack of a well assessed theoretical context to refer to. Thus, these results are interpreted in terms of qualitative arguments based on the chemical properties of the interactors in the given solvent medium.

## Introduction

Two dimensional materials (2DMs) have become one of the most explored areas of material science over the past decade because of their outstanding properties which have opened a way for an unparalleled scientific and technological number of applications. 2DMs are the ultrathin nanomaterials with high degree of anisotropy and chemical functionality (Chimene et al., 2015). The era of graphene (Gr) discovery, into single and few layer nanosheets (NSs) has sparked an intense research activity in almost all application areas covering optoelectronics (Ramanathan et al., 2008), catalysis (Guo et al., 2018), energy storage (Patchkovskii et al., 2005; Schedin et al., 2007; Dua et al., 2010), nano-biosensors (Kang et al., 2010; Gravagnuolo et al., 2015; Morales-Narváez et al., 2015, 2017; Cheeveewattanagul et al., 2017), polymer composites (Eda and Chhowalla, 2009), vast areas of biomedical studies (Ghosal and Sarkar, 2018) and many more. To unveil the potential of other 2DMs and to supplement the gapless feature of Gr, semiconducting 2D transition metal dichalcogenides (TMDs) such as MoS_2_/WS_2_ have been studied nicely because of their tunable band gap and crystal structure engineering. 2D TMDs held a great promise in electronics and optoelectronics because of their captivating properties. Besides this 2D TMDs have also been used in combination with Gr in applications such as field effect transistors (Late et al., 2012), energy storage (Yun et al., 2018), nano-biosensors (Yu et al., 2017), photo catalysis (Quinn et al., 2013; Hai et al., 2016) and biomedical sciences (Kaur et al., 2018), to name only a few (Wang et al., 2015; Sun and Wu, 2018) (Huang et al., 2013; Kong et al., 2013).

To utilize the 2D TMDs in biomedical applications, green and scalable production routes are critically required to understand their fate when in combination with living matter. Additionally, control over their production route will endow them with significant biocompatibility and tunable surface chemistry. Various fabrication techniques such as mechanical exfoliation (Ottaviano et al., 2017), epitaxial growth (Liu K. K. et al., 2012), hydrothermal and solvothermal production, ion intercalation (Zeng et al., 2011; Anto Jeffery et al., 2014; Zheng et al., 2014; Fan et al., 2015) and ultra-sonication have been employed to synthesize a broad family of 2DMs. Among the above cited fabrication methods, ultra sonication serves as an effective exfoliation strategy to obtain clean and defect free NSs where other methods failed due to low yield, expensive and complex equipment and intense use of harsh chemicals. Over the past years, substantial and remarkable efforts have been made toward the green production of TMD NSs (Nicolosi et al., 2013; Pan et al., 2016; Kaur et al., 2017b; Kaushik et al., 2018). Coleman and colleagues first utilized the liquid phase exfoliation (LPE), to be the most promising route to obtain clean and large scale production of monolayer and few-layer MoS_2_/WS_2_ NSs in numerous solvents by exploiting the concept of surface tension (Cunningham et al., 2012; Nicolosi et al., 2013; Backes et al., 2016b).

Kaur et al. (2017a) fabricated MoS_2_/WS_2_ NSs and MoS_2_-Gr hetero-structures by LPE in water-ethanol mixture. But the use of high boiling point and toxic solvents came up with a major difficulty in utilizing these solvents especially for biomedical applications. For instance, the presence of residual toxic solvent in the final samples, even in very low concentration, can impose adverse health impacts. According to the Hansen’s theory of solubility (Hansen, 2007), water is considered as a poor solvent and shows meager dispersibility of 2D TMDs. To overcome this trouble either with surfactant, polymer, enzyme or inorganic salt can be added to the solvent in sample fabrication to enhance the stability of 2D TMDs in water (Yao et al., 2013; Backes et al., 2014; Qi et al., 2015).

Interestingly, nano-objects of various types have demonstrated actions and impacts on live matter, there including both *in vitro* and *in vivo* cases. Apart from the extensive use of metal nanoparticles in antimicrobial studies, Gr and 2D TMDs have been widely studied so far to develop new technologies and novel antibacterial and antiviral nanocomposites to deal with the serious issues related to bacterial and viral infections. The considerable potential of MoS_2_ NSs in various biomedical applications also grasps its key role in synergistic antibacterial and antiviral action on various pathogens (Wu et al., 2016; Qu et al., 2017; Zhang et al., 2017, 2018, 2019; Kaur et al., 2018; Yuwen et al., 2018; Cao et al., 2019; Roy et al., 2019; Tang et al., 2020). The mechanism explained in the reported literature reveals a significant induction of physical damage and oxidative stress to the bacterial membrane which results in continuous disruption of bacterial cells and finally in cell death. As a result of these studies, MoS_2_ NSs turn out to be a better potential candidate than its derivatives in some cases, for the sake of bactericide and antiviral action.

The interactions of 2DMs with viruses has been less investigated, so far, as compared to that with bacteria. These studies mostly focused to date on Gr and graphene oxide (GO) samples tested on viruses. For example, recently nanoGr derivatives with polyglycerol sulfate and long alkyl chains have been tested in their interactions with HSV-1, with a highly time consuming fabrication protocol and multiple functionalization strategies (Rathinam et al., 2014; Deokar et al., 2017; Gholami et al., 2017; Donskyi et al., 2019). GO which is one of the most important graphene derivatives and a significant 2DM for various biomedical applications has been studied extensively for its novel antiviral action. Different multiple fabrication and functionalization routes have been adopted to exfoliate GO to enhance its antiviral action on different kind of enveloped and non-enveloped viruses (Sametband et al., 2014; Song et al., 2015; Ye et al., 2015; Chen et al., 2016; Cheng et al., 2017; Yang et al., 2017; Chekin et al., 2018; Du et al., 2018). In all of these cited research articles, modified Hummer’s method with complex functionalization and time consuming water based dispersion of GO nanosheets (GO NSs) have been employed.

To this aim, we have pushed our scientific efforts to knock at the door of 2DMs family to study the synergistic antibacterial and antiviral action of MoS_2_ to be compared with that of GO NSs, chosen as a reference material due to its documented bactericide and antiviral action (Sametband et al., 2014; Song et al., 2015; Ye et al., 2015; Chen et al., 2016; Cheng et al., 2017; Yang et al., 2017; Chekin et al., 2018; Du et al., 2018). Thus, here we tested the interactions of MoS_2_ and GO NSs with a gram-negative bacterium such as *Escherichia. coli (E. coli)* and with a gram-positive bacterium such as *Staphylococcus. aureus (S. aureus)*, in addition with the interactions with an interesting enveloped virus such as Herpes simplex virus type 1 HSV-1, responsible for widely spread infections to the face area (mouth and eyes), to the throat and sometimes to the central nervous system.

Thus, here we first fabricated GO and MoS_2_ NSs enhancing the stability of the nanoflakes using LPE in pure water with a careful optimization of the initial sonication parameters, such as initial concentration (*C*_i_), sonication time (*t*_s_), amplitude of the sonicator device (*A*_s_) and sonication vial. These parameters play a key role in defining the quality of exfoliation in various organic solvents, aqueous surfactant solutions and in pure water as well. We progressed in this way in the issue concerning the poor stability in water dispersed 2D NSs.

Produced dispersions were characterized by means of UV-Visible and Raman spectroscopy, zeta potential (ζ-potential) for surface charge analysis, scanning and transmission electron microscopies (SEM and TEM, respectively). For the morphological characterization of antibacterial and antiviral action of MoS_2_ and GO NSs on the given pathogens.

Very interestingly, we found a very different impact of both materials, MoS_2_ and GO, on bacteria and HSV-1.

As for bacteria, MoS_2_ showed a considerable bactericide effect in a short incubation time, 3–6 h, with both *S. aureus* and *E. coli*, whereas for GO the antibacterial action was lower and only began after 20 h incubation. Results in bacteria samples were interpreted in terms of the Derjaguin–Landau–Verwey–Overbeek (DLVO) theory (Thwala et al., 2013) that essentially accounts for the Liftshitz-van der Waals (LW) and the electrostatic interactions (EL), the latter due to the surface charge of bacteria on the one hand and of NSs on the other. By relying on a simple statistical approach, we could also estimate the probability per unit time for a bacterium, *S. aureus* or *E. coli*, to be killed by the damaging action of MoS_2_ NSs in our experimental conditions.

In order to evaluate the effect of MoS_2_ nanoflakes dispersed in water on HSV-1 we measured the infectivity inhibition possibly due to their interactions with viruses for four different experimental schemes: **virus** **pre-, co-treatment** with cells and viruses and **cell pre-** and **post-treatment.** To the best of our knowledge, the antiviral action of water-based dispersion of MoS_2_ NSs, i.e., fully biocompatible samples of this nanomaterial, on HSV-1 has not been reported so far in the literature. Moreover, thanks to our optimized fabrication technique, we could reach high concentration of NSs in our samples. GO NSs actual concentration in pure water was as high as 600 (GO-1) and 1400 (GO-2) μg/mL, which is consistently higher than that used be (Sametband et al., 2014) in similar experiments. As a result a strong antiviral effect in virus pre and co-treatment experiments with HSV-1 is found. MoS_2_ NSs concentration was in the range of 100–200 μg/mL, a concentration that turned out to be essential to observe antiviral effects in some of the co-treatment and virus pre-treatment experiments. Interestingly, the comparative impact of MoS_2_ and GO on HSV-1 was reversed with respect to what found in bacteria: while GO had a pretty strong antiviral effect in the virus pre-treatment and co-treatment experiments, MoS_2_ only induced some antiviral action in the virus pre-treatment case, while no antiviral effect was noted in either cell pre-treatment and post-treatment cases. The results found in viruses were interpreted in a more qualitative manner, since experiments with viruses are more complicated than with bacteria, due to the presence in the interaction of a third actor: the model Vero cell.

To summarize in a quick pictorial way our main experimental findings we report hereafter a table (Tables 1A,B, for bacteria and viruses, respectively) with a different color associated to each different interaction case going from hot red, for the strongest interaction, to lighter and lighter until white for the weakest looking like no interaction at all.

**TABLE 1A:** Summary of anti-bacterial actions of MoS_2_ and GO NSs on the tested pathogens, *S. aureus* and *E. coli* bacteria.

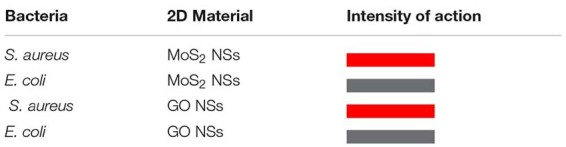

*The interaction of 2DM with both bacteria is associated with color code starting from hot red being the strongest interaction to lighter until white being the weakest interaction.*

**TABLE 1B:** Summary of anti-viral actions of MoS_2_ and GO NSs on the tested pathogens, HSV-1 DNA enveloped virus.

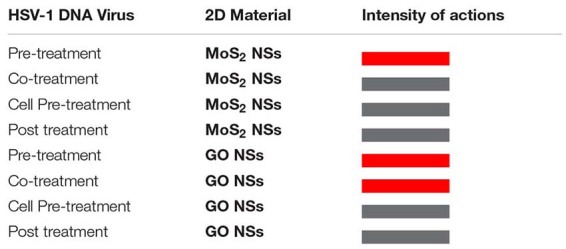

*The interaction of 2DM with the virus is associated with color code starting from hot red being the strongest interaction to lighter until white being the weakest interaction.*

## Results

### Synthesis of MoS_2_ NSs From Bulk Powder

To obtain a stable dispersion, our main focus was to carefully optimize the pre-sonication and post sonication parameters. In our previous article (Kaur et al., 2018), we were able to exfoliate MoS_2_ NSs in pure water with a good amount of stability to make the dispersions ready for various biomedical tests with live-matter. Then, inspired by Varrla et al. (2015) and Backes et al. (2016a, 2017), we adopted two-step exfoliation route with a motive to obtain good quality of 2D nanoflakes (particularly to avoid impurities), to enhance the final concentration and stability of the dispersion. It is worth to mention that apart from the sonication parameters, shape of the glass tube and of the probe, and minimum distance between probe and bottom of the tube used for exfoliation are of utmost importance to achieve highly stable dispersions. The latter case has been nicely explained by Santos et al. (2009) using the concept of dead zones for an efficient exfoliation. Immediately after the exfoliation the obtained dispersion was centrifuged at 2500 *g* (using Eppendorf Centrifuge 5810 R, Rotor F-34-6-38) for 90 min followed by the re-dispersion of the sediment into the fresh elix water of the same volume. Subsequently, a second step of exfoliation was carried out for longer duration (4 h) and at 55% amplitude. During exfoliation, the pulse mode was selected for 10 s on and 10 s off along with proper ice bath system to avoid the degradation of the exfoliated nanoflakes due to overheating. A careful check on the consistency of output power calculated from the energy obtained after every 40 min was maintained so to have an idea of the efficiency of exfoliation using the given parameters for longer duration. Furthermore, the fabrication and centrifugation parameters adopted for the exfoliation of GO NSs was different from that of MoS_2_. The corresponding details are reported in section “Materials and Methods.” Experimental details are reported in the Supplementary Material (SM).

### Material Characterization

The extinction spectra in the UV-visible region of MoS_2_ samples contain the contribution from both absorbance and scattering components. Both of these components are size dependent. In our centrifugation protocol at 40 and 160 g, the scattering component was dominant with high extinction peaks at 750–800 nm. At higher centrifugal forces, 1000, 2000, and 3000 g, the characteristic excitonic transitions are observed and the scattering background is reduced. In addition, the A-exciton peak shifted toward the lower wavelength region which is related to decreasing layer number. With the increase in centrifugal force, number of layers per flake decreases which results in few layered enriched dispersions. The corresponding UV-Vis spectra is shown in Supplementary Figures S1A–C together with the estimation of average number of layers, <*N*>, and lateral size, <*L*>, done through equations 1 and 2 in Supplementary Material (Backes et al., 2014), We notice that for GO the wavelength is read at 230 rather than 345 nm (Lai et al., 2012) as shown in Supplementary Figure S3.

### Raman Spectroscopy and ζ-Potential

Raman spectroscopy is a widely employed tool to estimate the thickness of TMD nanoflakes. Sample spatial homogeneity was tested via Raman micro-spectroscopy, by at least triplicating the Raman spectra of both MoS_2_ and GO (data not shown) in different sample spots. As for MoS_2_, the Raman spectrum shows two characteristic bands at$E_{2\,g}^{1}\,a\,n\,d\,A_{1\,g}$, which corresponds to the in-plane and out-of-plane vibrational modes, that for bulk fall at about 380 *cm*^–1^ and 403 *cm*^–1^, respectively. MoS_2_ nano-structuring modifies the Raman features of the bulk with an increase for the $E_{2\,g}^{1}$ frequency and a corresponding decrease of the *A*_*1g*_.

(1) $$\Delta \,\vartheta_{M\,o\,S\,2}=\vartheta_{A_{1\,g}}-\vartheta_{2\,g}^{1}$$

The frequency shift allows for an identification of the number of layers in the nanoflakes. the Raman spectra of MoS_2_ nanoflakes centrifuged at 2000 and 3000 *g* are shown, with laser excitation at 514.5 nm in Supplementary Figure S4. We observed similar modification in the Raman spectrum compared to bulk for both centrifugal protocols, with a common range of frequency shift Δν_MoS2_ of peaks ranging in the 23–24.6 *cm*^–1^ window. The Δν_MoS2_ range observed via Raman micro-spectroscopy corresponds to a nano-structuring spanning from 2 to 4 layers. These micro-Raman spectroscopy results look consistent with the range of nano-structuring indicated by UV-Vis extinction spectroscopy.

Generation of surface charges over the surface of 2D NSs plays a crucial role to understand the stability of LPE dispersions. To identify these surface charges, electrophoretic mobility measurements (*μ*) are performed. *μ* is proportional to the electric double potential around the charged nanoflake in the given solvent, the so-called ζ-potential (Gupta et al., 2015). In case of 2DMs, dynamic interactions among the NSs and their electrostatic stabilization play a fundamental role to anticipate the stability of liquid dispersions. It was observed that after the exfoliation, the MoS_2_ flakes exhibit high surface charge density depending upon the different centrifugal forces applied as seen from the Table 2. Generally, defect free MoS_2_ NSs are neutral and nanomaterial with no surface charge will precipitate in the end. Exfoliation of MoS_2_ NSs in water might have generated charged edges which were responsible for the stabilization of dispersed nanoflakes in pure water.

**TABLE 2:** ζ-Potential values of MoS_2_ NSs dispersion at different centrifugal forces.

**Centrifugal force (g)**	**Zeta potential *(ζ) mV***	**Electrophoretic mobility *(μ)***
620 g	−23.9 ± 0.6	−1.88 ± 0.04
1000 g	−25.6 ± 0.7	−2.01 ± 0.06
2000 g	−29.2 ± 1.3	−2.9 ± 0.1
3000 g	−23.4 ± 0.4	−1.84 ± 0.03

Raman spectra for GO NSs is reported in Supplementary Figure S5.

### Morphological Analysis of MoS_2_ NSs by SEM and TEM

**FIGURE 1:**
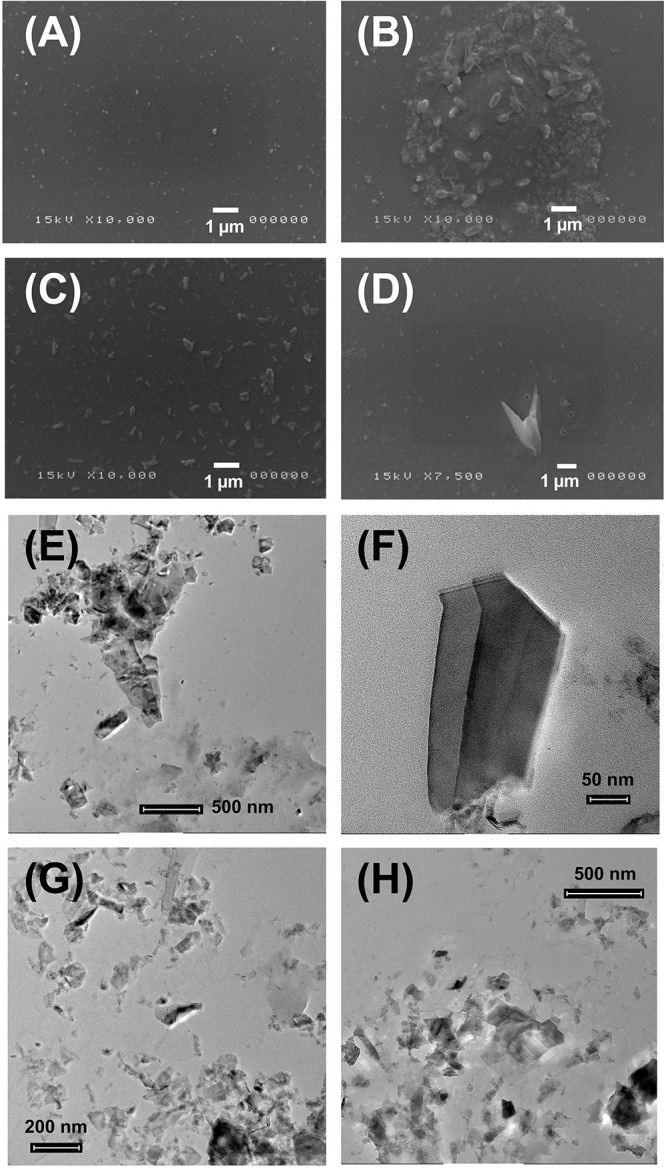
SEM and TEM representation of water dispersed MoS_2_ NSs at different resolutions (50 nm – 1 μm). **(A–D)** SEM analysis of MoS_2_-H_2_O dispersed NSs centrifuged at 2000 and 3000 g deposited on the gold sputtered silicon substrate. **(E–H)** TEM analysis of MoS_2_-H_2_O dispersed NSs centrifuged at 2000 and 3000 g deposited on the carbon grid. Scale bar for TEM is in the range of 50 nm – 500 nm and for SEM is 1 μm.

Exfoliation of 2D MoS_2_ NSs in water gave intriguing results in terms of its stability and morphology. Because of the poor solubility of MoS_2_ NSs in water it is very challenging to obtain a stable dispersion, resulting in a deposition of the dispersed 2D NSs onto substrates. Figures 1A–D represents the SEM analysis of water exfoliated MoS_2_ NSs centrifuged at 2000 *g* (Figures 1A,B) and 3000 g (Figures 1C,D). As it is clear in Figures 1A,B elongated clusters of 2D NSs are deposited. Sharp edged morphology with maximum density per unit surface was observed in the center of the given substrate used for deposition. In Figure 1C, randomly oriented nanoflakes having sharp-edges have been observed. In Figure 1D random deposition of small 2D NSs along with a very big sharp-edged nano-knife in the center are visible. Sharp-edges of 2D nanoflakes once in touch with biological membranes result in severe damage, either giving a puncture effect or a cut into the membrane.

We collected in Figures 1E–H TEM images of water exfoliated 2D NSs centrifuged at 2000 g (Figures 1E,F) and 3000 g (Figures 1G,H). Figure 1E shows few layer large ultrathin NSs with a wrinkled surface. A number of small NSs are placed onto the large chunk of NSs. In Figure 1F a very large and sharp-edged nanoflake is displayed. In Figures 1G,H a large number of NSs is visible, some of them being single layer. A chunk of multilayer NSs, aggregated in some portions of the deposited substrate, is due to evaporation with consequent aggregation.

### Antibacterial Efficacy of MoS_2_ NSs on *E. coli* and *S. aureus*: Experiment

*Escherichia coli* and *Staphylococcus aureus* were chosen as two microbial strains to investigate the antibacterial efficacy of directly water-exfoliated 2D MoS_2_ NSs at a range of concentrations. MoS_2_ NSs in general have been fabricated with an improved version of LPE, based on a two-step exfoliation route which enhances the overall stability and concentration of the final dispersion to render it suitable for various biomedical tests.

Both strains were used to evaluate the antibacterial capability of the nanostructure by broth micro-dilution method. The interaction of MoS_2_ NSs with the bacteria after culturing for 3 h, was observed by using SEM.

**FIGURE 2:**
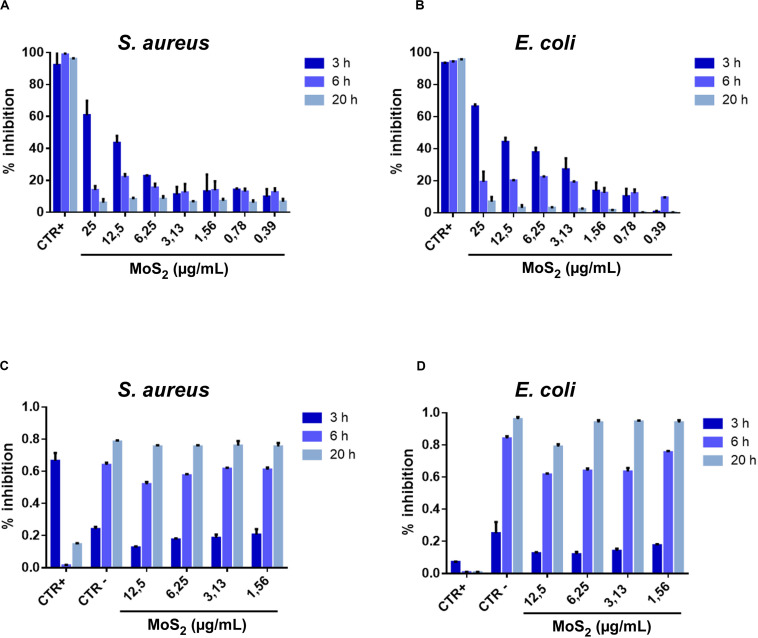
Percentage of bacterial growth inhibition after treatment with MoS_2_ NSs. **(A,B)** Histograms related to *S. aureus* **(A)** and *E. coli* **(B)** illustrate the antibacterial property of MoS_2_ NSs after interaction for 3, 6, and 20 h at various incubated concentrations. **(C,D)** Bacteria viability as measured by the optical density of the target at 600 nm (*OD*600) for different MoS_2_ NSs concentrations after interaction for 3, 6, and 20 h. **(C)** For *S. aureus* and **(D)** for *E. coli.* Ampicillin and vancomycin represented the positive controls for *E. coli* and *S. aureus*, respectively. The negative controls were consisted of deionized water. The data statistical error is standard deviation of three independent experiments. *P*-value is less than 0.05.

From the histograms shown in Figures 2A,B, where the bacterial growth inhibition (see section “Materials and Methods” for its calculation) is reported at various NSs concentrations and for 3, 6, and 20 h treatment duration, a significant antibacterial effect is observed at the concentration of 25 μg/mL. The antibacterial action of MoS_2_ NSs is due to mechanical lesions of the membranes of both bacteria (*E. coli* and *S. aureus*), as illustrated by the SEM images. In (A), the antibacterial effect decreases as the incubation time increases: at 12.5 μg/mL, the inhibition reaches 25%, remaining below 20% at higher concentration. At 20 h incubation, the antibacterial effect saturates and the inhibition growth reduces to 10%, with a substantial drop of the bactericide action. In (B), the antibacterial effect of 2D MoS_2_ NSs was a bit higher than for *S. aureus* in (A). At 25 μg/mL, a significant growth of the inhibition is visible which follows a decreasing trend with decreasing concentration when incubated for 3 h. At 6 h the antibacterial effect was in the 20-30% range, decreasing at low concentrations. At 20 h incubation, NSs are no more active.

Figures 2C,D represent the dynamic conditions used for the bacterial growth in presence NSs. Bacteria treated with NSs in Brain Heart Infusion liquid broth grow significantly, thanks to the appropriate nutrients.

We notice that, coherently with the measured inhibition reported in (Figures 2A,B), while we see an effect after 3 h incubation, this effect is nearly absent after 6 h and disappears completely after 20 h. This effect is modest because of the investigated low concentration range of MoS_2_ NSs in the dispersion and linearly scales with increasing the NSs concentration. For 3 h incubation the action is somewhat stronger for *E. coli* than for *S. aureus*, as shown in panels (Figures 2C,D).

### Antibacterial Efficacy of MoS_2_ NSs on *E. coli* and *S. aureus*: Discussion

The exposed sulfur layers of TMDs act as soft Lewis base which have strong affinity for metal ions. Exfoliated 2D TMD NSs undergo oxidative dissolution in a given aqueous media (Rodriguez et al., 2020). Membrane stress in atomically thin 2D NSs and high surface area to volume ratio have been accounted for the exposable cytotoxicity of 2D TMDs. Another reason for this toxicity is their aggregation which depends on features such as, thickness, surface area, and exposed edges (Mohona et al., 2019).

The DLVO theory analyzes the behavior of particles between forces, the principal ones being the van der Waals (vdW) force, typically attractive, and electrostatic repulsion (ER).

The net effect of two DLVO forces decides whether a 2D NM colloidal suspension will remain stable or not. The vdW force depends on the intrinsic properties of the material and the intermediate medium. The electrostatic force depends on acquired material properties, such as surface charge and radius, and other environmental parameters like electrolyte types, composition, and concentrations in the aqueous medium which can be twisted to tune colloidal interactions. The surface charge and distribution of associated counter-ions are sensitive to the nature and concentration of salts in the aqueous media. For a colloidal dispersion to be stable, the ER forces must be in large excess of the vdW.

### Antibacterial Efficacy of MoS_2_ NSs on *E. coli* and *S. aureus*: Time-Dependent Antimicrobial Effect

Interestingly, by measuring the bacterial inhibition it is possible to estimate the killing rate for a NSs of MoS_2_ to kill by damaging action a bacterium in the unit time, either for *E. coli* and *S. Aureus*, in our conditions of nanosheet and bacteria concentration and in our solvent medium (de-ionized water). By knowing this rat, ***k*_kill_**, one can also estimate the bacterium half-life, ***t^1/2^_kill_***, to half the bacterial population because of the NSs damaging effect, in case bacteria would not reproduce. In fact, by assuming no saturation effect for the bacterial growth and for NSs antimicrobial action, the growth rate of the bacterial population, ***N*(*t*)**, can be written as:

(2) $$\frac{d\,N}{d\,t}=k\,N\,\left(t\right)$$

where ***k*** is the overall rate of growth of bacteria and is given by:

(3) $$\text{k}={\text{k}}_{0}-{\text{k}}_{\text{kill}}$$

***k*_0_** and ***k*_kill_** being the population growth rate in absence of the NSs and the killing rate for a NS to kill a bacterium in the dispersion, respectively.

The inhibition, ***IN*(*t*)**, as a function of the incubation time, ***t***, can be written as:

(4) $$I\,N\,\left(t\right)=1-\frac{N\,\left(t\right)}{N_{0}}$$

where ***N*_0_** is the initial bacterial population. Then, from equations (2–4), integrating equation (2) with the initial condition ***N*(*t* = 0) = *N*_0_**, we can easily obtain for ***p*_kill_**:

(5) $$k_{k\,i\,l\,l}\,\left(t\right)=\frac{1}{t}\,\ln\left[\frac{1}{1-I\,N\,\left(t\right)}\right]$$

where we assumed the initial incubation time to be zero. Therefore, by measuring the inhibition versus the incubation time, ***IN*(*t*)**, we can estimate ***k*_kill_(*t*)** in the conditions of concentration of our experiments.

Thus, for instance, for ***t*** = 3 h and the highest concentration of MoS_2_ nanoflakes of 25 μg/mL, we obtain from Figure 2A:

(6) $$k_{k\,i\,l\,l}\left(t=3h\right)=\frac{1}{3\,h}\ln\left[\frac{1}{1-I\,N\,\left(3\,h\right)}\right]=\frac{1}{3\,h}\,\ln\left[\frac{1}{1-0.67}\right]\approx 0.367/h$$

where we have set ***IN*(*3*h)** ≈ 0.6/0.9 ≈ 0.67 at 25 μg/mL, since we have subtracted a background noise of about 10% from the positive control line as reported in Figure 2A. From equation (6) we can estimate the bacterium half-life, ***t^1/2^_kill_***, namely the time it takes for the bacterial population to be halved because of NSs damaging, in case bacteria would not reproduce. We obtain, then:

(7) t=k⁢i⁢l⁢l1/2ln⁡2kk⁢i⁢l⁢l≈1.89h≈113minutes

meaning that in approximately a couple of hours incubation time the *S. aureus* population would be halved due to the antimicrobial effect of MoS_2_ NSs, in our experimental conditions. This time should be compared with the bacterial doubling time that characterizes their growth, which is much shorter both for *S. aureus*, approximately 24 min (Domingue, 1996) in the typical laboratory conditions such as in our experiments, and *E. coli*, in which case it is reported to be 20 min (Gibson et al., 2018). For a NSs concentration of 25 μg/mL the average number of MoS_2_ NSs per mL is approximately 2.2 × 10^11^, whereas the initial number/mL of bacteria in our culture is ***N*_0_** = 5 × 10^5^. Then, given the above bacteria doubling time, it is straightforward finding that in natural growth conditions, with no nanoflakes, the bacteria population per mL would reach 2.2 × 10^11^ in about 454 and 381 min for *S. aureus* and *E. coli*, respectively. We stress here that our model is just a first approximation of the real kinetic of bacteria culture growth in the presence of the MoS_2_ NSs that damage bacteria, since in our simplified model we decouple the bacteria population number from the number of available nanoflakes. This latter number is not constant, but rather decreases with time, since nanoflakes that interact with bacteria and damage them most likely will then stick on the damaged bacterium outer membrane, due to the attractive forces that led to that interaction. Thus, the nanoflakes that interacted with a bacterium basically do not have to be considered available anymore for further interactions and, because of that, the actual number of nanoflakes per unit volume diminishes with time. However, this decrease is much slower that the bacteria growth rate. In our simplified vision we do not take this interplay into account, but we can anyway provide a first interpretation of the observed kinetic effect in the MoS_2_ nanoflake antimicrobial action. Then, based on the above considerations, the interpretation of Figure 2 is clear: the maximum antimicrobial action is observed after 3 h of incubation for both *S. aureus* and *E. coli*, whereas just a little effect is seen after 6 h of incubation and nearly no inhibition is measured after 20 h. In fact, while after 3 h the number of NSs per unit volume is still pretty higher than the number of bacteria and this leads to a tangible bactericide action, around 6 h of incubation the number of bacteria per unit volume passed the value of 2.2 × 10^11^, corresponding to the initial number of MoS_2_ nanoflakes, due to the faster bacterial growth in comparison with the decrease of the available MoS_2_ nanoflake population. Thus, bacteria are growing more and more with the increase of the incubation time, whereas available nanoflakes are only decreasing, leading to the observation at 20 h where no appreciable antimicrobial effect is detected.

### Antibacterial Efficacy of MoS_2_ NSs on *E. coli* and *S. aureus*: Interaction Energy Calculation

In our case, surface interactions of ultrasonically exfoliated MoS_2_ NSs with both gram negative and Gram positive bacteria shows interesting results. The active sites of 2D MoS_2_ nanoflakes form a bridge between the surface S atoms and surface compounds on bacterial cell membrane. The presence of different functional groups on *E. coli* and *S. aureus* provides a strong interaction with the surface atoms for the first 3–4 h which resulted in bacterial cell death and disruption of its cell membrane (Zhang et al., 2016). Based on the DLVO theory (Thwala et al., 2013), we can estimate the interaction energy between bacteria and MoS_2_ nanoflakes, similarly to what recently done to describe the interaction between bacteria strains and gold nanoparticle (Pajerski et al., 2019). Thus, we here generalize the DLVO theory to 2D nano-objects, such as nanoflakes. Within the DLVO theory applied to interacting bacteria and nanoflakes, being suspended in aqueous solution, the total interaction energy, *V*^tot^, can be written as (Israelachvili, 2011; Hwang et al., 2012; Ohshima, 2014; Pajerski et al., 2019):

(8) $$V^{t\,o\,t}=V^{E\,L}+V^{V\,W}$$

being:

(9) $$V^{E\,L}=\frac{\pi \,a_{1}\,a_{2}\,\left(\varsigma_{1}^{2}+\varsigma_{2}^{2}\right)}{a_{1}+a_{2}}\{\frac{2\,\varsigma_{1}\,\varsigma_{2}}{\varsigma_{1}^{2}+\varsigma_{2}^{2}}\ln\left[\frac{1+\exp\left(-k\,d\right)}{1-\exp\left(-k\,d\right)}\right]+\ln\left[1-\exp\left(-2kd\right)\right]\}$$

the electrostatic interaction energy of a bacterium having an effective radius *a*_1_ and ζ-potential *ζ*_1_ positioned at a distance *d*, into a solution having permittivity *ε*, from a nanoflake having an effective radius *a*_2_ and z-potential *ζ*_1_, and

(10) $$V^{V\,W}=-\frac{A\,a_{1}\,a_{2}}{6\,d\,\left(a_{1}+a_{2}\right)}$$

the LW interaction energy between the two nano-objects. Please, notice that *d* refers to the separation distance between a bacterium and a nanoflake, namely it stands for the bacterium membrane-nanoflake distance, where the bacterium is an extended object having an average size itself, often of the order of magnitude of *d*. The *k* constant in equation (9) is the inverse Debye-Hückel length, *λ*_D,_ namely the average radius of the sphere in the solvent medium around a charged object beyond which there is no electrostatic interaction anymore because of the shielding effect of the surface charge by the ions present in the solution. It essentially depends on the solution pH, but also on the solvent medium permittivity and temperature as:

(11) $$k=\frac{1}{\lambda_{D}}=\sqrt{\frac{2\,z^{2}\,e^{2}\,n}{\varepsilon_{r}\,\varepsilon_{0}\,k_{B}\,T}}$$

where *z* is the charge of the ion *e* = 1.6 × 10^–19^ C the elemental charge, *n* the ion density, *ε*_r_ the solvent medium relative permittivity (80 in our case for a water-based solution), *ε*_0_ = 8.854 × 10^–12^ C^2^/J.m the vacuum permittivity, *k*_B_ the Boltzmann constant and *T* di absolute temperature of the solution. In our case, for a slightly basic pH of 7.4, at 20°C we can estimate (Israelachvili, 2011) a Debye–Hückel length *λ*_D_ ≈ 2 μm.

The constant *A* in equation (10) is the so-called Hamaker constant, that takes into account the van der Waals body-body interaction coupling, and for two nanosized objects interacting into a dispersion can be defined as (Ohshima, 2014):

(12) $$A=\left(\sqrt{A_{1}}-\sqrt{A_{d}}\right)\,\left(\sqrt{A_{2}}-\sqrt{A_{d}}\right)$$

where *A*_1_, *A*_2_ refers to the only bacterium and the only MoS_2_ nanoflake, respectively, in a dry condition, i.e., when their bacterium-bacterium and nanoflake-nanoflake van der Waals interaction is not mediated by a solvent medium and *A*_d_ refers to the dispersion medium to account for its internal van der Waals interaction strength. Thus, in our case, for the interaction between bacteria and MoS_2_ nanoflake we can calculate *A* = 1.25 × 10^–20^ J, where we used for *A*_1_ = 5.2 × 10^–20^ J for both bacteria species (Farahat et al., 2009), *A*_2_ = 29.6 × 10^–20^ J for MoS_2_ nanoflakes (Mohona et al., 2019) and *A*_d_ = 3.7 × 10^–20^ J for water-based solvent (Israelachvili, 2011).

As for the geometrical parameters *a*_1_ and *a*_2_ we adapted the DLVO theory, ideally developed for spherical objects, to our nanosized interactors, bacteria and nanoflakes. The calculated effective radii, *a*_1_ and *a*_2_, were the radii of a sphere having the same volume of the real average volume of the considered partner. Hence, we obtain *a*_1_ = 360 nm and 630 nm, for *S. aureus* and *E. coli*, respectively, and *a*_2_ = 134 nm for MoS_2_ nanoflakes, where the *S. aureus* radius is directly obtained as the average size of the SEM bacterial images since this bacterium is spherical, but the effective radius of *E. coli* and MoS_2_ nanoflake are obtained as the radius of a sphere having the same volume of a cylinder with 2.1 μm height and 0.4 μm base radius, for *E. coli* and NSs having 100 nm average lateral size and 1 nm average thickness for MoS_2_ nanoflakes, as retrieved by our nanoflakes characterization reported in Figure 1, respectively.

Then, by using equation (10) and equation (12) for a separation distance of about *d* = 0.16 nm, which corresponds to the case when bacterium and nanoflake are in touch with a contact, short-range interaction, what is the case when damaging effect takes place (Bayoudh et al., 2009; Pajerski et al., 2019), we obtain for the Van der Waals term:

(13) $$V^{V\,W}=\begin{array}{c} -25.99\times 10^{-20}\,J=-64.96\,k_{B}\,T\,f\,o\,r\,S.a\,u\,r\,e\,u\,s\\ -26.22\times 10^{-20}\,J=-65.55\,k_{B}\,T\,f\,o\,r\,E.c\,o\,l\,i \end{array}$$

where the minus sign indicates that the interaction is attractive and the reference to the thermal energy is made for 20°C where *k*_B_*T* = 25*meV*.

As for the electrostatic interaction term in equation (9), given the short distance when nanoflake and bacterium are in touch, *d* = 0.1 nm, then *kd* ≈ 5 × 10^–5^ and we can safely assume exp(-*kd*) ≈ 1-*kd* and exp(-2*kd*) ≈ 1-2*kd*. We can assume from the literature *ζ*_1_ = −37.1 mV and −12.7 mV for *S. aureus* and *E. coli*, respectively (Oh et al., 2018). We do observe that, despite both negatively charged, the two bacteria species have pretty much different ζ- potentials, which lead to a different value and even sign of the electrostatic interaction when they are in touch with a nanoflake. This behavior can be ascribed to the different structure of the bacteria cell membrane, gram-positive for *S. aureus* and gram-negative for *E. coli*, resulting in a different nature and distribution of charged and polar groups on the bacterial membrane, The cell wall of *S. aureus* involves layers of peptidoglycans that are rich in teichoic acid groups (Lovering et al., 2012; Romaniuk and Cegelski, 2015; Oh et al., 2018). Then, the considerably high measured negative value of the ζ- potential for *S. aureus* is ascribed to the existence of anionic phosphate groups in the glycerol phosphate repeating units of teichoic acids (Brown et al., 2013). On the hand, the outer layer of *E. coli* contains mainly lipopolysaccharides (Maurer et al., 1999; Brown et al., 2015), which include phosphate groups in the inner core and polar hydroxyl groups in sugar repeating units of the *O*-antigen (Frirdich and Whitfield, 2005; Ho and Waldor, 2007). Phosphate and hydroxyl groups can account for negative ζ- potential of *E. coli* and also for its observed hydrophilicity. As a result, the electrostatic interaction of *E. coli* with negatively charged MoS_2_ nanoflakes, mediated by a slightly basic water-based environment can turn to attractive at short distances as *d* = 0.16 nm in our case. Therefore, based on the above considerations, by means of equation (6) we can calculate the electrostatic contribution to the interaction energy when nanoflakes and bacteria are in touch, namely the electrostatic contribution to the binding energy for a contact interaction to be:

(14) $$\begin{array}{c} V^{E\,L}=\begin{array}{c} 5.09\times 10^{-20}\,J=12.7\,k_{B}\,T\,f\,o\,r\,S.a\,u\,r\,e\,u\,s\\ -2.59\times 10^{-20}\,J=-6.475\,k_{B}\,T\,f\,o\,r\,E.c\,o\,l\,i \end{array} \end{array}$$

We observe that the electrostatic contribution to the binding energy is smaller than the vdW, which is a surface energy term and, interestingly, it changes sign being positive for *S. aureus* and negative for *E. coli*. Finally, from equations (13) and (14) the overall interaction energy for a contact interaction between bacteria and MoS_2_ nanoflakes, namely an estimate for the binding energy, is found to be:

(15) $$V^{t\,o\,t}=\begin{array}{c} -20.9\times 10^{-20}\,J\approx -52.26\,k_{B}\,T\,f\,o\,r\,S.a\,u\,r\,e\,u\,s\\ \begin{array}{c} -28.81\times 10^{-20}\,J\approx -72.25\,k_{B}\,T\,f\,o\,r\,E.c\,o\,l\,i \end{array} \end{array}$$

Thus, we estimate that in our conditions the binding energy between MoS_2_ nanoflakes and *E. coli* is about 40% larger than that for *S. aureus*, what might explain a somewhat higher inhibition effect for *E. coli* as compared to *S. aureus*. We also notice that for both bacteria strains negative values, i.e., energies due to an overall attractive net force of interaction, in the 50–70 *k_B_T* is in the same range as those found for the interactions of other bacteria strains, such as *Bacillus subtilis* and *Staphylococcus carnosus*, with gold nanoparticles, the adsorption of which requires between 120 and 170 *k_B_T*. Certainly, in our case it is important noting that we are neglecting any form factor for the interacting nano-objects and assuming they are spheres with an effective radius: while this assumption is surely verified for *S. aureus*, it is certainly not as valid for *E. coli*, which is shaped as a rod, and for MoS_2_ NSs, which are 2D NSs.

As for the behavior of the bacteria inhibition with the concentration of MoS_2_ NSs, it appears substantially linear, as seen in Figures 2A,B for both *S. aureus* and *E. coli*., especially after 3 h incubation, when the inhibition effect of the nanoflakes is evident. However, we notice that while for *E. coli* a consistent effect is present already for a concentration of 3.13 μg/mL, in the range of ≈ 30% inhibition, for this concentration in *S. aureus* the measured inhibition is only ≈ 10%, which is in practice in the background of the signal (see the positive control bar of the histogram in Figure 2A, refereed to 3 h incubation, that reaches ≈ 90% with an error bar of ≈ 10%). It seems that for *S. aureus* there is concentration threshold-like effect: below the 3.13 μg/mL we see nearly no inhibition. In *E. coli*, instead, such a threshold is absent, falling perhaps at concentration values lower than the minimum in our measurement, 0.39 μg/mL. This effect may be due to a different critical volume around the bacterial cell, where the attraction forces dominate. This critical volume, that can be different for *S. aureus* and *E. coli*, is defined by a critical radius, *r*_crit_ (Pajerski et al., 2019) and may results in the difference in the inhibition at the intermediate concentrations between the two bacterial strains, giving this threshold-like behavior for *S. aureus*. In fact, a too small critical volume around *S. aureus* might lead to no availability of nanoflakes in that volume with a consequent drop in the inhibition effect. This mechanism can, of course, only represent qualitative explanation of the threshold-like behavior for *S. aureus*, for two reasons: (i) the available number of NSs per critical volume is dynamical since bacteria population is growing while the number of available NSs is decreasing with time as they are kept by the interaction with some damaged bacterium and (ii) NSs and bacteria move with time in space with respect to each other while they are in suspension, under the effect of diffusion and the relative interaction forces. This latter effect determines the critical volume to move as well in time through the suspension. However, certainly a consistent difference in the critical volume, with a value much smaller for *S. aureus* than for *E. coli*, would be an argument in favor of the observed behavior.

**FIGURE 3:**
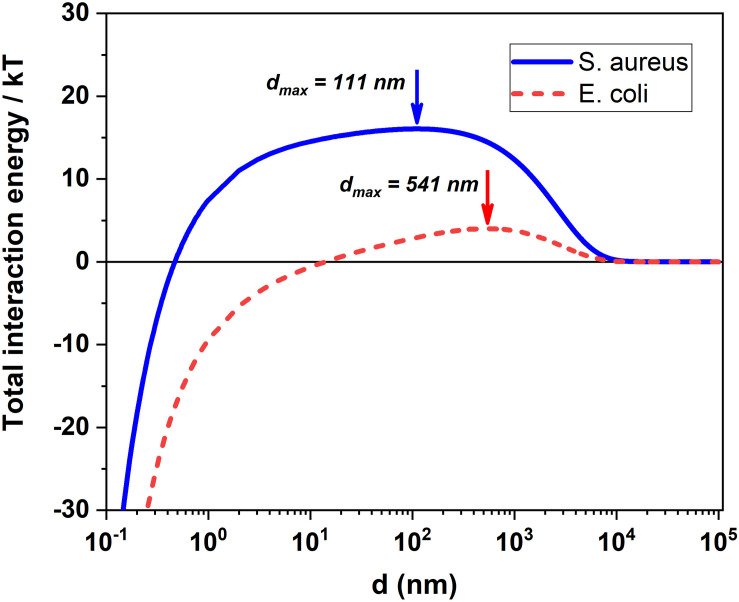
DLVO prediction for the total interaction energy between MoS_2_ NSs and *S. aureus*/*E. coli* in our experimental condition. Total interaction energy between the bacteria and MoS_2_ NSs and d (nm) indicates the location of energy maxima for both *E. coli* and *S. aureus* at 541 nm and 111 nm.

Then, we remind that *r*_crit_ is defined as (Pajerski et al., 2019):

(16) $$r_{c\,r\,i\,t}^{3}\approx {\left(d_{\max}+a_{1}\right)}^{3}-a_{1}^{3}$$

where *d*_max_ indicates the location of energy maxima in DLVO profile for each bacterial species of radius *a*_1_ (see Figure 3), i.e., the value of the separation, *d*, between bacteria surface and NSs for which the potential energy barrier is encountered. The value of *d*_max_ is easily found from equation (8) by imposing $\frac{\partial }{\partial d}\,V^{t\,o\,t}\,\left(d\right)=0$ and solving for *d* the subsequent equation:

(17) $$\frac{A}{d^{2}}+\frac{12\,\pi \,\varepsilon \,k\cdot e^{-k\,d}}{1-e^{-2\,k\,d}}\cdot \left[\left(\zeta_{1}^{2}+\zeta_{2}^{2}\right)\cdot e^{-k\,d}-2\,\zeta_{1}\,\zeta_{2}\right]=0$$

which is a transcendental equation. However, by neglecting in equation (17) the LW force term *A*/*d*^2^ which is very small in the *d*-range where the potential energy barrier falls (see Figure DLVO), we can work out a simple and nice analytical approximated formula for *d*_max_:

(18) $$d_{\max}\approx \frac{1}{k}\cdot \ln\left(\frac{\zeta_{1}^{2}+\zeta_{2}^{2}}{2\,\zeta_{1}\,\zeta_{2}}\right)=\lambda_{D}\cdot \ln\left(\frac{\zeta_{1}^{2}+\zeta_{2}^{2}}{2\,\zeta_{1}\,\zeta_{2}}\right)$$

The values of *d*_max_ numerically calculated by finding the precise position of the potential energy barrier (see Figure 3) are *d*_max_ = 111 nm and 541 nm for *S. aureus* and *E. coli*, respectively, in excellent agreement with those calculated by our analytical formula given in equation (18), that read 102 nm and 536 nm, respectively. This means that $\frac{r_{c\,r\,i\,t}^{3}\left(E.coli\right)}{r_{c\,r\,i\,t}^{3}\left(S.aureus\right)}\approx 23$ what may well explain the observed presence of a concentration threshold in *S. aureus*, threshold which is absent in *E. coli*.

A number of considerations still are interesting and useful for longer term bacteria-MoS_2_ nanoflakes interactions. The solubility of water exfoliated MoS_2_ NSs in pH = 7.4 also gives an additional stability to the dispersed 2D nanoflakes which enhances their interaction with the pathogen. Generally, the presence of salts in a given solvent reduces the stability of exfoliated 2D NSs because of the charge screening effect. This directly affects the electrophoretic mobility of the given ions in the medium. The absolute values of μ decrease more with the increase in divalent ion concentration than with increase in monovalent ion (i.e., Na^+^) concentration as the latter (i.e., Ca^2+^) screen the electric double layer and induce a smaller *λ*_D_ than the former. Also, specific adsorption of divalent cations along with the implication of short ranged attractive non-DLVO forces have also been considered as one of the reasons for the reduction of diffuse layer potential (Wu et al., 2018). At a specific critical concentration of given salts, avoids the nanoflakes to aggregate. Addition of salt with concentrations greater than the critical coagulation concentration leads to sharp increase in aggregation over time. With the increasing mono- and divalent (NaCl and CaCl_2_) salt concentrations, the surface charge on the 2DMs gets screened thereby accelerating aggregation. After 20 h of incubation, the excessive multiplication of bacteria over the time screens MoS_2_ nanoflakes and reduces its ability to affect its cell membrane. This in turn could be related to decreasing stability in the given culture medium, rich of nutrients and suitable for bacterial growth. With time, MoS_2_ NSs get stacked to each other forming a multi-layered structure which results in fewer active sites available for a strong interaction with bacterial membrane.

### Antibacterial Efficacy of GO NSs on *E. coli* and *S. aureus*: Experiment and Discussion

As for the interaction of GO NSs with the two bacterial strains, we should notice, first, that we could access in this case higher GO NSs concentrations than for MoS_2_, given the good hydrophilicity of 2D GO (Zeng et al., 2019).

**FIGURE 4:**
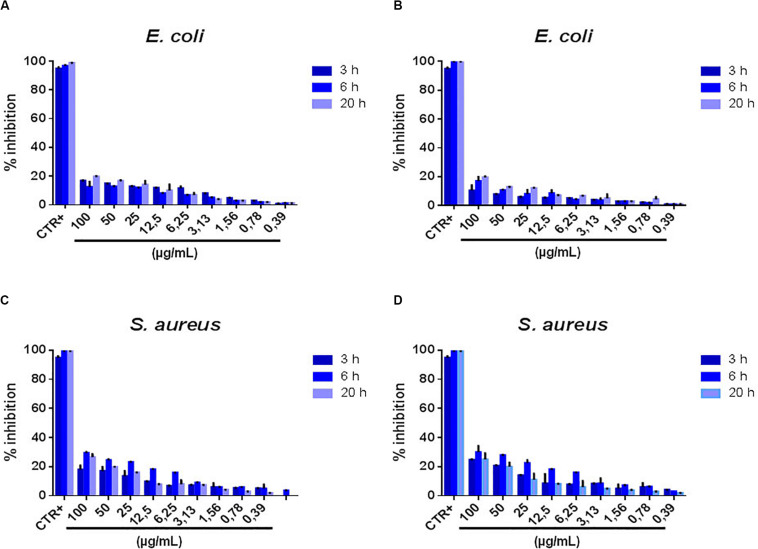
Percentage of bacterial growth inhibition after treatment with GO NSs. Panel **(A,B)** Percentage inhibition *E. coli* at 3-6-20 h of incubation with GO NSs (600 μg/mL & 1400 μg/mL). Panel **(C,D)** Percentage inhibition *S. aureus* at 3-6-20 h of incubation with GO NSs at same concentration. Ampicillin and vancomycin represented the positive controls for *E. coli* and *S. aureus*, respectively. The negative controls were consisted of deionized water. The data statistical error is standard deviation of three independent experiments. *P*-value is less than 0.05.

As a general feature our results in this case show a much lower inhibition as compared to MoS_2_, even at the very high concentration of 100 μg/mL. This is true mostly for shorter incubation times, namely 3 and 6 h. Some more antimicrobial effect is present in *E. coli* at 20 h. Another general feature is the linear increase of the antimicrobial action with the GO NSs concentration. However, the inhibition never exceeds ≈ 20% in *E. coli* after 20 h incubation at 100 μg/mL GO NSs concentration for both the initial concentrations of the nanoflakes preparation of 1400 [GO(1)] and 600 μg/mL [GO(2)] and never exceeds ≈ 30% in *S. aureus* after 6 and 20 h incubation at 100 μg/mL GO NSs concentration for GO(2) as shown in Figures 4A–D. In this case, by exfoliating two initial preparations of NSs having different concentration, GO(1) and GO(2), we could study the interaction of bacteria with GO NSs having different average sizes. In fact, for GO(2) the average lateral size and thickness of the GO NSs are ≈ 400 nm and ≈ 1.5 nm, respectively, whereas for GO(1) average lateral size and thickness result ≈ 200 nm and ≈ 1 nm, respectively. Then the effective radius, *a*_2_, of the GO NSs results ≈ 21.1 nm and 38.6 nm for GO(1) and GO(2), respectively.

By following the approach developed in the previous section in equations (8)–(12), we calculated the interaction energy for bacteria-GO NSs when they are in touch, separated by a distance *d* = 0.1 nm. We can look at this energy as the interaction binding energy of a single GO nanoflake interacting with a bacterium. We find that these energies, calculated by the DLVO theory and reported in Table 3, are positive for *S. aureus*, indicating an overall repulsion at short distances with GO NSs, while they are negative for *E. coli*, indicating instead an attraction. We also find that these contact interaction energies, regardless the sign, are smaller for smaller nanoflakes, as it makes sense. Thus, the result for *S. aureus* indicates that other mechanisms of action are at fundament of the observed modest antimicrobial effect, differently from what observed for MoS_2_ NSs.

**TABLE 3:** Interaction energies for a separation distance *d* = 0.1 nm between GO 2D NSs and *S. aureus* and *E. coli* bacteria, calculated by the DLVO theory.

**(A)**	***V*^VW^ (in 10^–20^ J)**	***V*^ET^ (in 10^–20^ J)**	***V*^tot^ (in 10^–20^ J)**	**(B)**	***V*^VW^ (in 10^–20^ J)**	***V*^ET^ (in 10^–20^ J)**	***V*^tot^ (in 10^–20^ J)**
*S. aureus*	9.67	31.1	40.77	*S. aureus*	5.54	17.84	23.38
*E. coli*	10	−43.6	−33.6	*E. coli*	5.6	−24.2	−18.6

***(A,B)** Refer to 600 and 1400 μg/mL concentration of the initial GO nanoflake preparation, respectively, corresponding to an effective nanoflake radius, *a*_2_, of 38.6 and 21.1 nm, respectively.*

For *E. coli* we found negative contact energies that implies attractive forces, though it is reported that for the specific interaction of GO 2D nanoflakes with the *E. coli* membrane are predominantly repulsive and that this repulsion occurs between deprotonated carboxylic acid groups in GO and negative cell membrane of bacteria (Palmieri et al., 2017). However, an attraction between the two interactors is due to the presence of divalent cations in the medium and the functional groups on the surface of GO. This reduces the repulsion and favors collisions between GO NSs and bacteria (Palmieri et al., 2017). As a result, of these observations, we have to hypothesize that also for *E. coli* the mechanism of the modest measured antimicrobial effect of GO nanoflakes is to ascribe to actions other than damaging contact interaction between a single nanoflake and a bacterium.

Certainly, two important factors such as the nature of the bacteria strain, whether gram positive or gram negative, and the specific bacterial membrane can play a role in the observed antimicrobial action. Polymer peptidoglycan, a major component of the bacterial cell walls, in both gram positive and gram negative bacteria, has a different polymeric layer structure in either species. The large multilayer region of peptidoglycan with a wall teichoic acid (WTA) and lipoteichoic acid (LTA) in gram positive bacteria with thick cell walls are attached to the peptidoglycan layer and to the cell membrane, respectively. Whereas, the gram negative bacteria have a thin layer of the peptidoglycan, in the region called periplasmic space, situated between the cell and outer membranes. Instead of teichoic acids, the gram negative bacteria synthesize lipopolysaccharides (LPS), which form distinct areas within the outer phospholipids bilayer (Malanovic and Lohner, 2016; Pajerski et al., 2019).

Collisions between nanoflakes and bacteria, driven by attractive forces, are largely dependent on the stability of GO in broth. Generally, due to charge screening effect, a reduced antibacterial efficacy is observed, especially for the first 3–6 h incubation, before GO aggregates formation will take place (Romero-Vargas Castrillón et al., 2015). In fact, though GO is highly dispersible in pure water, the presence of different salts and nutrients as in broth reduces its stability and leads to fast aggregation of the exfoliated nano flakes (Akhavan et al., 2011), what occurs in 12–24 h incubation, by reaching aggregates having a typical linear size in the 1.5–3 μm (Palmieri et al., 2017). Once formed GO aggregates interact with both bacterial strains, with stronger affinity with gram positive than with gram negative bacteria, due to the presence of functional groups on the former type bridging with different functional groups on GO.

Moreover, over longer incubation time, with the formation of large GO aggregates a second mechanism of action of GO, the so-called wrapping (Liu S. et al., 2012), can bind bacteria and inhibit their growth. Particularly, in this wrapping effect, bacteria are insulated from the external environment with a reduction in movement and proliferation (Geesey et al., 2000). The wrapping is most efficient for Gram positive *S. aureus* may be due to specific cell wall architecture (Akhavan et al., 2011). In a given broth, divalent cations tend to wrap bacteria more than monovalent.

Due to the size and shape the wrapping mechanism by GO 2D nanoflakes aggregates of *E*. *coli* takes longer than for *S. aureus* and is less effective. In fact, the longer size of *E. coli*, namely the height of the rod, is ≈ 2–3 μm, whereas the *S. aureus* sphere radius is ≈ 0.4–0.5 μm. That means that the spatial hindrance of *E. coli* is much bigger than that of *S. aureus*, resulting also in a much larger surface: about 6.5 versus 1.5–1.6 μm^2^. As a result, we can expect the wrapping mechanism for inhibition to be a bit more effective in *E. coli* than in *S. aureus*. In fact, for instance, at a concentration of 100 μg/mL we see in Figures 4A–D after 20 h incubation an inhibition of ≈ 20% for *E. coli* against ≈ 30% for *S. aureus*.

### Antibacterial Action by SEM Characterization

**FIGURE 5:**
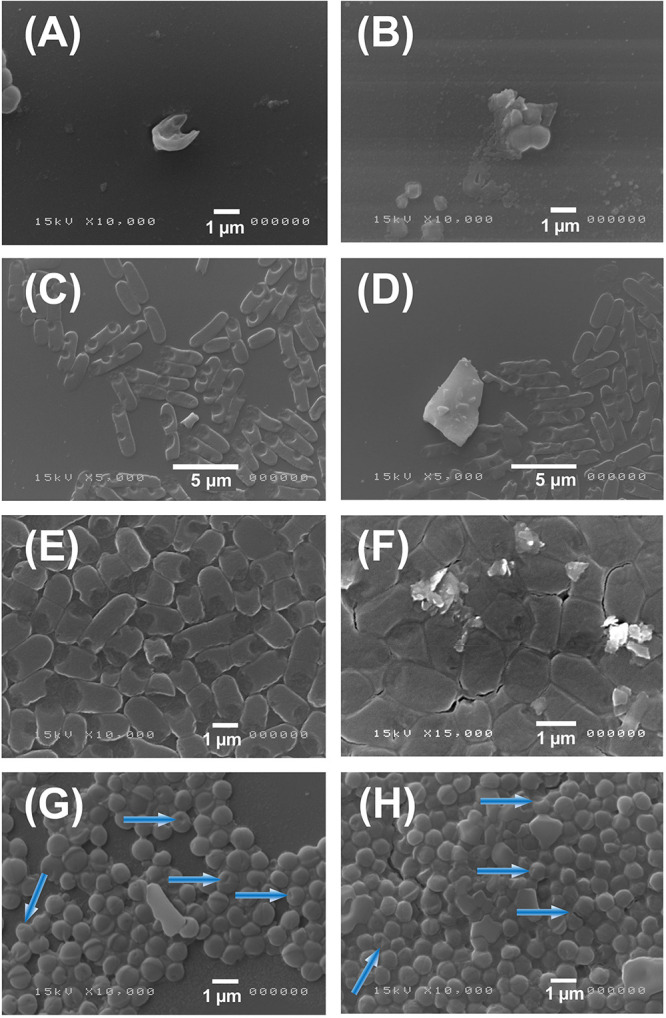
SEM images of *S. aureus* and *E. coli* after treatment with MoS_2_ NSs. Panel **(A–D)**: SEM images of *S. aureus* **(A,B)** and *E. coli* **(C,D)** illustrate the antibacterial effect of MoS_2_NSs after interaction for 3 h. Panel **(E–H)**: SEM images of *E. coli* **(E,F)** and *S. aureus* **(G,H)** illustrate the antibacterial effect of MoS_2_NSs after interaction for 6 h and the subsequent damage shown in blue arrows.

In order to monitor the antimicrobial action, we characterized by SEM analysis the interaction of MoS_2_ nanoflakes with both *S. aureus* and *E. coli* bacteria as shown in Figures 5A–H. Images reported in Figures 5A–D shows 3 h incubation of MoS_2_ NSs with both bacteria. In the Figure 5A,B panels we show typical images of *S. aureus* that interacted with MoS_2_ NSs: in Figure 5A we see a single damaged *S. aureus* bacterium, whereas in Figure 5B, we show the damage caused to *S. aureus* with the leakage of its cellular components. The presence of some sharp-edged flakes nearby resulted in the fragmentation of the bacterium and the subsequent bacterial death.

In the Figures 5C,D panels we show images of *E. coli*. We first notice the typical rod-like shape having a height of ≈ 2 μm and a base of ≈ 1 μm diameter along with the affected ones. *E. coli* rods are often damaged, as observed in the bacteria where a piece is missing in the rod, like a bite, or in some other cases they are more severely damaged being fragmented as shown in Figure 5C. Whereas, in Figure 5D a big flake is evident with many other smaller flakes on the top. We notice the typical lateral size of MoS_2_ NSs to be much smaller than ≈ 0.5 μm, as seen in Figure 5C, whereas in Figure 5D, we notice the damages induced by the big flake having a lateral sizes between 2 and ≈ 4–5 μm to be particularly strong, with bacterial fragments all around, likely due to the sharp-edges of this big flake.

The Figures 5E,F panels refer to *E. coli*, whereas Figures 5G,H report *S. aureus* images incubated for 6 h. Firstly, we can notice by eye the damage is less evident and less frequent, in agreement with the inhibition measurement shown in Figures 2A,B and the consequent analysis as shown in blue arrows. The bacterial density is also much increased as compared to the (A–D) panels, since bacteria had longer time to multiply. The presence of flakes and their aggregation degree is also reduced as compared to 3 h incubation time. We also notice that the amount of heavily damaged bacteria, nearly destroyed or fragmented as seen in (A–D), is less.

It is possible to quantify the interaction of nanomaterial with *E. coli* and *S. aureus* counting the bacteria damaged and not damaged (Table 4). Table 4 shows the number of bacteria damaged and not damaged presents in two or three images representative of samples and their respective percentage.

**TABLE 4:** Shows percentage of damaged and not damaged bacteria when incubated with MoS_2_ NSs.

***E. coli***				***S. aureus***		
	**Damaged**	**Not Damaged**	**% Damaged**	**Damaged**	**Not Damaged**	**% Damaged**
Figures 5C,D				Figures 3A,B		
3 h incubation	64*	11*	85%	2*	/	100%
3 h incubation	38*	7*	84%	2	/	100%
Figure 5E				Figures 3G,H		
6 h incubation	55*	3*	91%	86*	41	68%
6 h incubation	46*	2*	96%	90*	50*	64%

It represents the statistical data which quantifies the interaction of MoS_2_ NSs with *E. coli* and *S. aureus* for 3 and 6 h incubation. *Image with MoS_2_ flakes.

It is evident that in *E. coli* sample the interaction with MoS_2_ is greater than in *S. aureus*, with a damage percentage value between 83 and 99%, while in *S. aureus* this value is between 57 and 79%. The difference in the damage percentage between NSs and *E. coli* and *S. aureus* is on the average 25%, what can be ascribed to a different morphology of these bacteria. The damaging action on the spherical morphology of *S. aureus* is more difficult than on the rod-like shape of *E. coli*. This is also enlightened in our model where we calculate a lower total interaction energy for *S*. *aureus* than for *E*. *coli* (see Equation 15) by about 28%, which is in very good agreement with the statistical observation based on SEM images. This is a consequence of the smaller effective radius of the former bacterium as compared the latter. Generally, in the images in which MoS_2_ flakes appear the percentage of damaged bacteria is higher.

### Antiviral Effect

**FIGURE 6:**
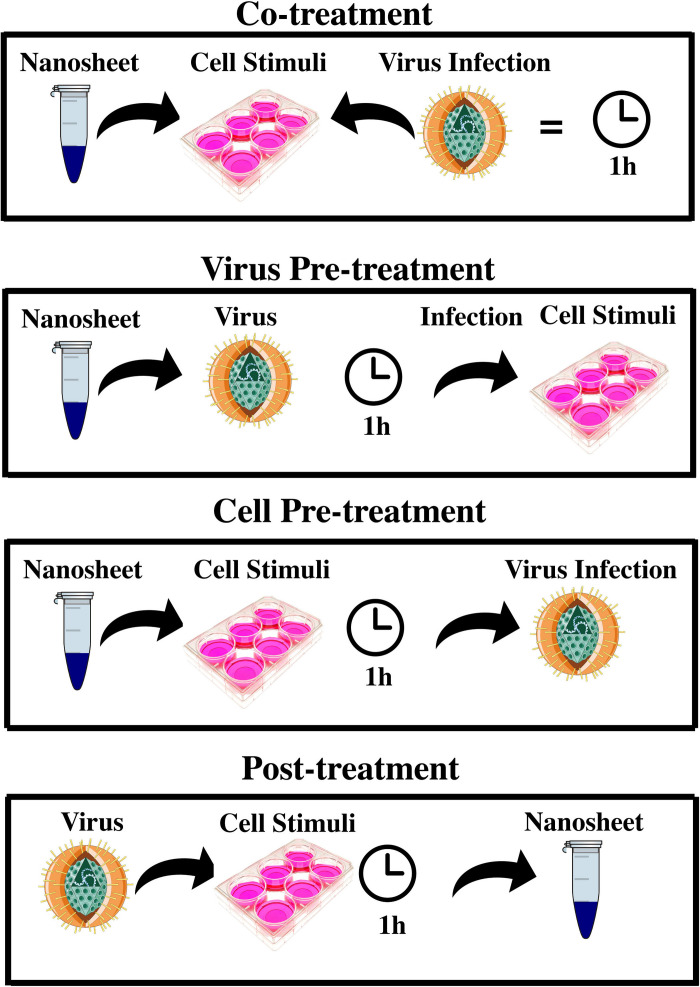
Different schemes of experimental antiviral assay. It shows the different interaction schemes of 2D MoS_2_ and GO with HSV-1 and Vero cells exhibiting very intriguing results in either cases (2D MoS_2_ and GO NSs).

To measure an antiviral effect by MoS_2_ and GO 2D NSs on HSV-1, we measured the infectivity inhibition induced in a Vero cells model by four different experimental schemes: virus pre-treatment, co-treatment, cell pre-treatment and post-treatment as shown in Figure 6. Pre-treatment assay was performed through two different approaches, that for readiness we call virus pre-treatment and cell pre-treatment in the manuscript. In the former, the virus was first incubated with nanoflakes by 1 h and then added to Vero cells, a renal epithelial cell line from *Cercopithecus aethiops*, for another 1 h incubation. In cell pre-treatment, cells were first treated with nanoflakes by 1 h and then infected with HSV-1. In the co-treatment, viruses and nanoflakes were delivered together to the cells and incubated for 1 h, whereas in the post-treatment Vero cells, incubated with HSV-1 for 1 h, were later added with nanoflakes for another 1 h incubation. After inactivation of non-penetrated viruses by citrate buffer (pH 3.0), cell monolayers were incubated at 37°C in Minimum Essential Medium (MEM) supplemented with 5% carboxymethylcellulose. After 2 days, the cells were fixed with 4% formaldehyde and stained with 0.5% crystal-violet, and the plaques were counted. The experiments were performed in triplicate and the percentage of viral inhibition was calculated compared to the untreated HSV-1 control (CTR-). In all the four schemes we tried different concentrations of the NSs, for GO material we tried also the effect of a different NSs lateral size coming from two initial different preparations.

The general scenario of the obtained results is very interesting and somewhat surprising. As a general feature we found that in our experiments GOs were more potent antiviral agents than MoS_2_ NSs contrary wise than for the bactericide action. Essentially, we summarize our results in this way: (i) in the virus pre-treatment case we observed a moderate antiviral action by MoS_2_ nanoflakes in comparison with a more robust effect by GO NSs; (ii) in the co-treatment case, the most surprising and intriguing finding in our investigation about antiviral actions, no effect was observed by MoS_2_ nanoflakes in comparison with a strikingly strong effect by GO NSs, an antiviral action even stronger than for the virus pre-treatment case; (iii) no antiviral action was observed at for both MoS_2_ and GO nanomaterials in the cell pre-treatment; and (iv) post-treatment cases.

We verified that none of the two nanomaterials has a cytotoxic action itself on the Vero cells, what is shown in Supplementary Figure S5. Their 50% cytotoxic concentration (CC50) is higher than 100 μM. Cytotoxicity was evaluated through MTT [3-(4,5-dimethylthiazol-2-yl)-2,5-diphenyltetrazolium bromide] assay performed on Vero cells that interacted with MoS_2_ (A) and GO (B) showing no change in the cell viability for all the tested concentrations.

We interpret our results in viruses on the base of qualitative arguments, differently from the case of bacteria, since experiments in viruses end up with a sample much more complicated to deal with, due to the interaction with a cell model to account for, together with the direct interaction between NSs and viruses. Thus, quantitative or semi quantitative models, finding a solid context within a well assessed physico-chemical theory such as DLVO as for bacteria-nanoflake interactions, do not exist to the best of our knowledge. In addition, the surface interaction of MoS_2_ NSs with viruses and the related cell model such as Vero cells has never been investigated so far to the best of our knowledge, as well as the antiviral activity of bare MoS_2_ NSs in water-based systems.

### Virus Pre-treatment Experiment

**FIGURE 7:**
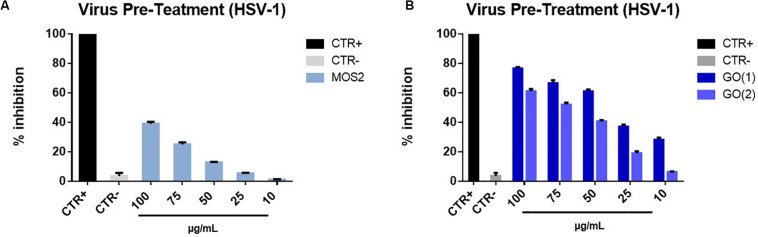
Virus pre- treatment with MoS_2_ and GO NSs. HSV-1 infectivity inhibition with MoS_2_ **(A)** and GO NSs **(B)**, of two different types GO(1) and GO(2), having different lateral size and thickness. Not infected and not treated cells represented the positive control (CTR+), meanwhile infected cells were the negative control (CTR–). The data statistical error is the standard deviation of three independent experiments. *P*-value is less than 0.05.

In the virus pre-treatment experiment we did observe an antiviral effect by both nanomaterial, though more evident for GO, as reported in Figure 7. For MoS_2_ NSs, that have an average later size of ≈ 150 nm with an average thickness of ≈ 1.2 nm, we reached a maximum inhibition of about 40% for the highest NSs concentration of 100 μg/mL, whereas the antiviral action reaches its maximum at about 75 and 65% inhibition for 100 μg/mL concentration for the two different types of GO NSs, GO(1) GO(2), respectively. The 50% effective concentration (EC50), that refers to the concentration at which there is 50% viral infection inhibition, was 40.57 μM for GO(1) and 68.32 μM for GO(2). We notice that the two different initial concentrations correspond to different average lateral size and thickness of the GO nanoflakes, being the average lateral size ≈ 175 nm and ≈ 500 nm, and the average thickness ≈ 1.2 nm, ≈ 2.5 nm, for GO(1) and GO(2), respectively.

We ascribe the less effective antiviral action of MoS_2_ as compared to that of GO to the formation of −SH groups on the MoS_2_ flake surface, mostly in correspondence of the edge of the flakes due to the exfoliation procedure for fabrication. It is known, in fact, that in LPE fabrication sulfur atoms concentrate mainly on the edge of the flake and they are subsequently functionalized during H_2_O cleavage process occurring in the fabrication method, with the consequent formation of −SH groups (Rodriguez et al., 2020). On the other hand, it is also well known that thiol groups are present in the cysteine amino acid, abundant in many spike glycoproteins of viruses. For example, in the case of HSV-1 the B Glycoprotein, essential for the attachment and fusion to the host cell (Cooper and Heldwein, 2015; Franci et al., 2017; Stelitano et al., 2019) has a cysteine residue (Laquerre et al., 1998) that can certainly repel with thiols, which can be formed on the MoS_2_ flake edge, where the abundant presence of sulfur atoms in solution can lead to the formation of −SH groups with the capture of an H^+^ ion.

Furthermore, we notice that (i) for both MoS_2_ and GO NSs the lower the concentration the higher the inhibition with a linear increase and (ii) that, in case of GO, the smaller nanoflake, GO(1), is more effective that the bigger flake, GO(2), likely due to the presence on its surface of sharper edges (Hu et al., 2013; Szabo et al., 2020).

Additionally, we notice that the stability of GO nanoflakes in water-based dispersion is higher than that of MoS_2_ NSs to corroborate a higher antiviral impact of the former compared to the latter.

### Co-treatment Experiment

**FIGURE 8:**
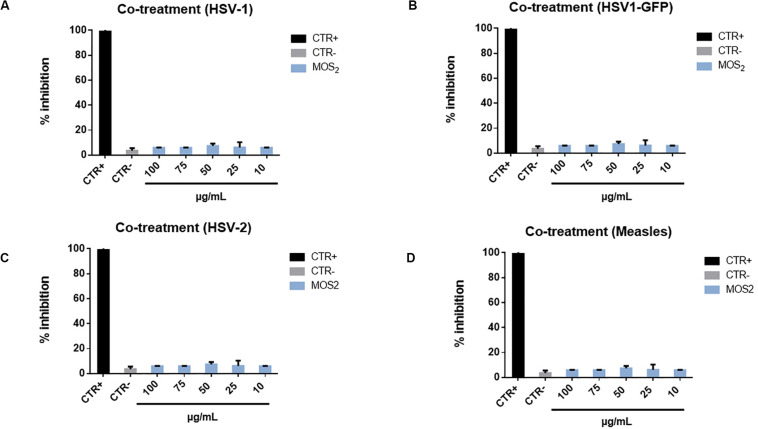
Co-treatment with MoS_2_ NSs and HSV-1-GFP, HSV-2 and Measles virus. Infectivity inhibition after treatment with MoS_2_ nanosheets and HSV-1 **(A)**, HSV-1-GFP **(B)**, HSV-2 **(C)**, and measles virus **(D)**. No antiviral action was detected for any of the tested nanosheet concentrations. Not infected and not treated cells represented the positive control (CTR+), meanwhile infected cells were the negative control (CTR–). The data statistical error is the standard deviation of three independent experiments. *P*-value is less than 0.05.

In the co-treatment experiment we obtained pretty intriguing and somewhat unexpected results. In the case of MoS_2_ NSs we did not observe any antiviral effect. Then, to corroborate this negative result we carried on further tests. We extended the inhibition experiment to other two viruses, i.e., Herpes simples virus type-2 (HSV-2) (which is mostly associated to genital herpes, whereas HSV-1 has a prominent oral localization), and Measles virus. The results are reported in Figure 8 are however, negative: no antiviral action is observed.

An additional test was performed through which we double checked this negative response by infecting Vero cells with another strain of HSV-1 virus, where the Green Fluorescent Proteins (GFP) has been inserted into the genome sequence into the gene encoding VP22 tegument protein, so to make this virus fluorescent in the green band. Then by measuring the amount of green fluorescence emitted by Vero cells, while growing infected with this virus, it is possible to monitor the eventual antiviral action or the inhibition. This is reported in Supplementary Figure S6 for various concentrations of the MoS_2_ NSs, where no antiviral action is present in none of the analyzed cases as a consistent green fluorescence confirms.

We explain this finding based on the same argument used in the previous subsection to motivate the minor efficacy of MoS_2_ NSs in the virus pre-treatment case as compared to GO NSs. But rather, in the co-treatment experiment the argument is much reinforced. In fact, the MoS_2_ NSs can likely be functionalized in the medium by acquiring protons, i.e., H^+^ ions, on their edges rich of sulfur atoms content, thus forming thiol groups. These groups then are highly repelled by the Vero cell membranes, which have −HS groups on their surface (Sametband et al., 2014). Essentially, the mechanism is similar to what described for the virus pre-treatment case, but much more efficient now, MoS_2_ nanoflakes are strongly repelled and going to the opposite direction. Eventually, no antiviral action is measured.

**FIGURE 9:**
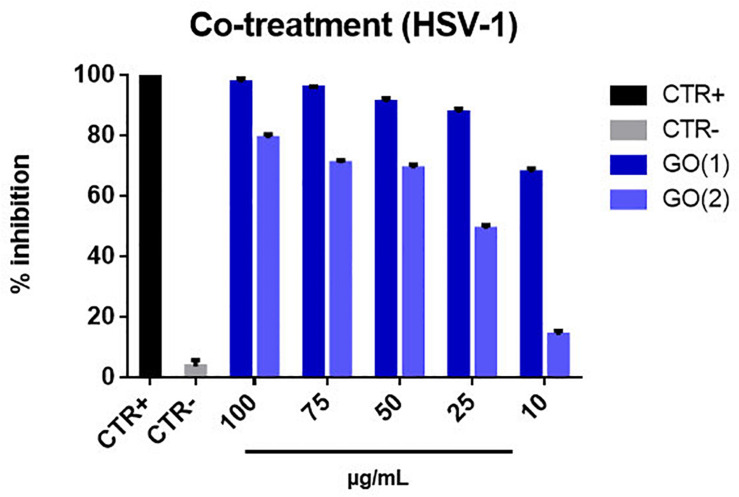
Co-treatment with GO NSs. The graph shows the percentage of inhibition of viral infection after treatment with GO [GO(1) and GO(2)]. We remind that for GO(1) the average lateral size of the nanoflake is ≈175 nm whereas for GO(2) is ≈500 nm, the average thickness being ≈1.2 nm and ≈ 2.5 nm, for GO(1) and GO(2), respectively. Cells and cells infected with virus represented the positive and negative controls, respectively. The data statistical error is the standard deviation of three independent experiments. *P*-value is less than 0.05.

As for GO nanoflakes the co-treatment experiment leads to an impressive and somewhat unexpected very strong antiviral action, even stronger that for virus pre-treatment. Indeed, we measured inhibition of the order of 90–100% for concentrations in the 25–100 μg/mL of nanomaterial for GO(1) and a highest inhibition of ≈ 80% for GO(2), as reported in Figure 9. We ascribe this intense action to the presence of specific glycoproteins on the Vero cell membrane like Lectin, that have high affinity with negative groups present on the surface of GO NSs in solution, given the presence of electrolytes, such as carboxyl and epoxy (Klukova et al., 2016). This implies a high probability for both GO NSs and HSV-1 to meet and be in touch in correspondence of the Vero cell membrane, a kind of additional indirect interaction mediated by the presence of Vero cells. Then this mechanism adds to the direct interaction between nanoflakes and viruses, responsible for the antiviral action observed in the pre-treatment case, to reinforce the overall antiviral effect.

Moreover, we notice that the antiviral impact decreases with decreasing GO concentration; in particular, this decrease is linear in case of GO(2), but for GO(1) we may assume that the decrease effect is just saturated at concentrations higher than 25 μg/mL, being the inhibition already as high as ≈90%. We also comment about the higher impact observed for smaller nanoflakes, GO(1), as compared to bigger nanoflakes, GO(2): in fact, smaller nanoflakes are characterized by sharper edges and a consequent stronger antiviral power than bigger NSs.

### Cell Pre-treatment and Post-treatment

**FIGURE 10:**
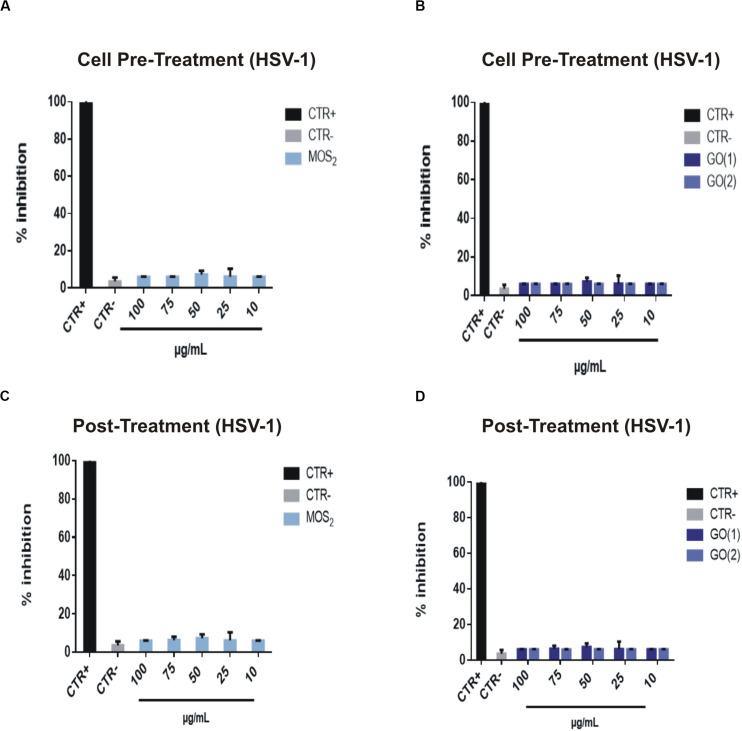
Cell pre-treatment and post-treatment assay after treatment with MoS_2_ and GO NSs. Vero cells were treated with MoS_2_ **(A)** and GO NSs **(B)**, and then infected with HSV-1 in the cell pre-treatment assay. Infectivity inhibition experiment in the post-treatment scheme. **(C)** After addition of MoS_2_ NSs, **(D)** or treatment with GO NSs. No antiviral activity was reported for both NSs in the two antiviral experiments. Cells and cells infected with virus represented the positive (Acyclovir) and negative controls, respectively. The data statistical error is the standard deviation of three independent experiments. *P*-value is less than 0.05.

Figures 10A–D panel shows two different schemes of interaction: cell pre-treatment and post-treatment. In the former case, cells were incubated with 2D NSs for 1 h followed by the addition of the viruses for 1 h incubation. In the latter case, cells were firstly incubated with HSV-1 for 1 h; after this first step, 2D NSs were added contemporarily by virus removal from the given mixture for 1 h incubation. As we can see from the (A), the antiviral action is in this case low, just above the uncertainty in measuring zero action, represented by the negative control bar. In fact, the percentage of inhibition using 2D MoS_2_ NSs is less than 10% at each tested concentration. We can interpret this effect as due to the repulsion between MoS_2_ NSs and Vero cell membrane because both have negative surface charge. In (B), the result of the cell pre-treatment experiment is reported for GO. The antiviral effect is small also in this case for both GO(1) and GO(2) and concentration independent. This is likely due to the weak interaction of GO with the cell membrane together with the fast aggregation of GO in the broth medium in the presence of different ions and other nutrients, regardless the NSs size and thickness.

In the post-treatment experiment we did not see antiviral effect in any case, either with MoS_2_ and with GO NSs, as shown in (C,D). With MoS_2_ the effect was completely absent, whereas with GO it was nearly vanished, i.e., lower that ≈ 10% inhibition for both types of nanoflakes. We interpret this result as the affinity of viruses with Vero cells is so high that in the first 1 h incubation where the nanoflakes have not been added yet, nearly all the viruses present in the broth entered cells. Once the nanoflakes are added they cannot exert a direct damaging action onto the viruses as free viruses into the medium are not available anymore. On the other hand, nanoflakes are not capable to enter Vero cells as they are too big. Therefore, they can only approach the cell membrane, but as shown in Supplementary Figure S5 both nanomaterials have been proven not to be cytotoxic, thus their action on the cells is substantially negligible.

### Antiviral Action Characterization by TEM

**FIGURE 11:**
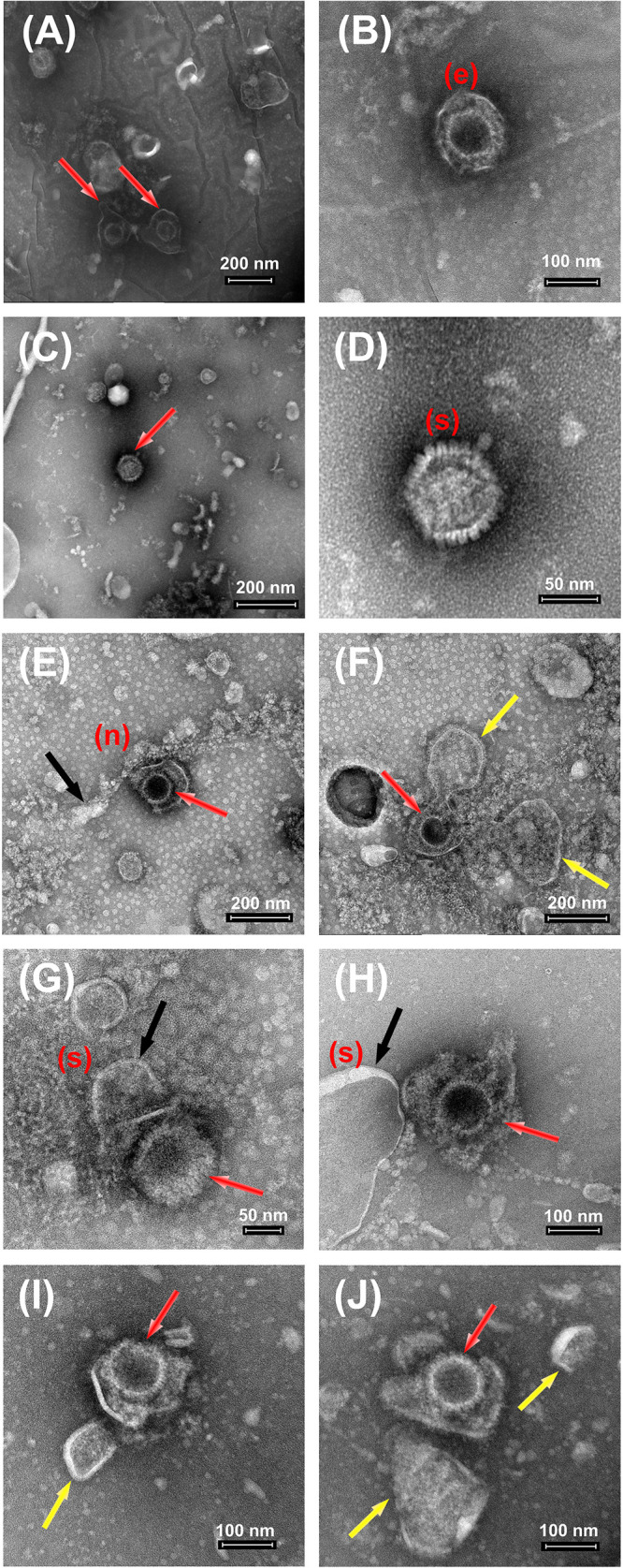
TEM images of HSV-1 after treatment with MoS_2_ and GO NSs. **(A–D)** Control HSV-1 at 10^9^ PFU/mL. The above panel **(A–D)** shows the control images of HSV-1 with its intact spherical structure indicated with red arrows (in **A,C**). The unaffected virus structure has been nicely imaged showing the outer envelope (e) in **(B)** and spikes (s) in **(D)** over its surface. **(E–H)** The above panel **(E–H)** shows the control images of HSV-1 at 10^9^ PFU/cell and MoS_2_ NSs at 100 μg/mL. HSV-1 is indicated with red arrows, meanwhile NSs with black ones. The yellow arrows indicate the opening and destruction of viral envelope MoS_2_-inducted. NSs are shown as a chain (n) or real sheets (s) around the virus surface affecting its membrane. **(I,J)** Control HSV-1 at 10^9^ PFU/cell and GO NSs at 50 μg/mL (initial preparation concentration 1400 μg/mL). HSV-1 is indicated with red arrows, meanwhile GO NSs with yellow ones. All bright field TEM images have been acquired upon negative staining treatment with phosphotungstic acid solution (2%).

In order to visualize and better characterize the damaging action onto HSV-1 viruses by nanomaterials, both MoS_2_ and GO NSs, we carried out TEM analyses of samples prepared in the pre-treatment scheme as shown in Figures 11A–J. Here below we report a few examples of images of only viruses as viewed by TEM (Figures 11A–D), of viruses interacting with MoS_2_ (Figures 11E–H) and GO nanoflakes (Figures 11I,J) after the first hour of incubation before Vero cells are added.

The panel (A–D) represents the morphological features of only HSV-1 after some purification steps and then deposited onto the given substrate. As we can see from (A), three main parts are visible: (a) the central position of the core; (b) a layer called capsid surrounding the core; and (c) an envelope surrounding the capsid as shown by red arrows. Usually, the capsid comes in different angular positions and it remains stable in the given medium. In (B), we can see an envelope, indicated by (e) in the panel, enclosing a capsid. The outer boundary of this layer appears to be denser than the remaining structure. Whereas, in (C-D), we can see spikes as covering the outer layer of the HSV-1, indicated by (s) in the (D) panel that is a magnification of (C). These spikes mark the presence of some glycoproteins present on the virus surface for the attachment to the Vero cell membrane.

The panel in (E-H) shows the presence of water dispersed 2D MoS_2_ NSs in black arrows, while red arrows indicate viruses. As it can be seen from (E), MoS_2_ nanoflakes are organized in a chain, indicated as (n), around the virus surface affecting its membrane. The edges of the flakes are in contact with the virus envelope which induced opening of the virus envelope and led to reduced vitality in the given medium. In (F) we did observe right the cut in the virus envelope by a nanoflake with the consequent leak of the internal material of the virus. In fact, we see that the viral envelope of the virus of is no more in its spherical shape: two rounded wide areas of leaked viral material are visible on both sides of the nanoflake chain, highlighted by yellow arrows, evidencing the virus disruption. Due to negative staining effect, that helps to enhance the virus contrast, a clear morphology of MoS_2_ NSs is not visible, as our focus was to look for damaged virus structure. However, concentration dependent antiviral action is observed: in the condition of the images, in fact, we measured a slight virus inhibition. Another contact interaction is imaged in (G) and (H). Here, we can clearly see the presence of MoS_2_ nanoflakes in black arrow (indicated as s in the (A-D) and the subsequent disruption of virus surface with no more outer envelope. In (H), a large size MoS_2_ nanoflake with a marked sharp edge oriented toward a virus is shown.

GO in our case has shown pretty much intriguing results depicting its strong and evident antiviral action. Here red arrow indicates viruses and yellow arrows GO flakes. The above images show the presence of wrinkled GO NSs present near the virus membrane. The sharp edges of small size nanoflakes in this case, GO(1), have affected the virus envelope of HSV-1 resulting in the disruption of proteins present on its surface, as seen for two virus-flake couples in (I-J). The presence of glycoproteins on HSV-1 carrying positive charge attracts the negative surface charge of graphene oxide and a specific orientation of GO NSs with its sharp edges disrupts its membrane leading to virus significant inhibition.

## Conclusion and Prospects

In summary, we have reported a significant improvement in the fabrication of MoS_2_ NSs by achieving a considerable amount of stability and concentration in pure water as a solvent. Thanks to the LPE technique which gives access to tune various exfoliation parameters such as *C*_i_, *t*_s_, *A*_s_ and sonication vial. These parameters play a key role in defining the quality of exfoliation in various organic solvents, aqueous surfactant solutions and in pure water as well. Apart from MoS_2_, fabrication of GO in pure water with a very high initial concentration (600 and 1400 μg/mL) and thickness in the range of 1.2 nm −2.5 nm has been achieved. Very interestingly, we found intriguing antibacterial and antiviral action of both GO and MoS_2_ NSs on the tested *E. coli* (gram negative), *S. aureus* (gram positive) bacteria and HSV-1. In particular, MoS_2_ showed a considerable bactericide effect in a short incubation time, 3–6 h, with both *S. aureus* and *E. coli*, whereas for GO the antibacterial action was lower and only began after 20 h incubation. Particularly, to explain the obtained antibacterial results we developed a refinement of models based on the DLVO theory, considering the role of electrostatic and vdW interactions in the attachment efficacy of a given 2DMs with the bacteria under study. We have also estimated the probability per unit time for a bacterium (*S. aureus* or *E. coli*) to be killed by the inhibition action of MoS_2_ NSs. This antibacterial action reduces after 20 h of incubation due to the fast multiplication rate of the bacterium whereas MoS_2_ NSs are all taken by contact interactions with already damaged bacteria. On the other hand, GO showed completely different results exhibiting its antibacterial action after 20 h of incubation which we have ascribed to the so called ‘wrapping mechanism,’ due to large aggregates of GO NSs formed because of to the presence of different electrolytes in the given broth. The results found in viruses samples were, instead, interpreted in a more qualitative manner, since interactions with viruses are more complicated than with bacteria, due to the presence of a third interacting species: the Vero cells host model. We measured the infectivity inhibition induced in a Vero cells model by four different experimental schemes: virus pre-treatment, co-treatment, cell pre-treatment and cell post-treatment. Surprisingly, the impact of MoS_2_ and GO on HSV-1 virus was reversed as compared to the actions on bacteria: while GO had a pretty strong antiviral effect in the virus pre-treatment, that only detects the direct NSs -virus interaction, and co-treatment experiments, MoS_2_ only induced some antiviral action in virus the pre-treatment experiment. No antiviral effect was noted in either cell pre- and post-treatment case for both nanomaterials. The very interesting GO co-treatment case has puzzled the scenario because direct interaction of GO with virus is pretty strong: we interpret this as due to the presence of specific glycoproteins on the Vero cell membrane that have high affinity with the oxygen functionalized groups on the GO NSs surfaces, such as carboxyl and epoxy. This results into an increased affinity of GO NSs with the Vero cell membrane, where NSs and viruses certainly have the highest likelihood to meet and to be put in contact interaction. Our findings open very interesting prospects both (i) to understand the role of specific broth constituents and their chemical properties in view of GO and MoS_2_ NSs functionalization, when interacting with bacteria and viruses, and (ii) also exciting perspectives of applications given the specific antibacterial and antiviral observed actions. In forthcoming experiments, we aim at studying also how the interactions of 2D NSs impact on genetic sequences of interacting viruses, to possibly unveil some of the interaction pathways. For instance, it could be worth in the next studying gene regulation in macrophages in response to viral and bacterial infections. It is known, in this case, that TRIM29 regulates the activation of alveolar macrophages, inducing the expression of type I interferons and proinflammatory cytokines in alveolar macrophages (Xing et al., 2016) and DNA virus infection (Xing et al., 2017). It would be interesting to study the effect of 2D NSs on the expression of TRIM29 for the control of viral and bacterial lung infections.

## Materials and Methods

### Statistical Methods for Data Analysis

As for the characterization of the fabricated MoS_2_ and GO NSs, results of spectroscopic and stabilization measurements as well as morphological analysis are always the average of 3–5 acquisitions for each single scan. In particular, UV-visible spectra for MoS_2_ and GO, as shown in Supplementary Figures S1–S3, have been recorded three times for each scan. For Raman spectra, each scan has been recorded five resulting in the average shown in Supplementary Figures S4, S5. As for the ζ-potential shown in Table 2, the standard deviation values are calculated over 5 scans per sample, giving the reported uncertainty on the best estimated value (average).

For SEM images statistical analysis, we have calculated the percentage of damaged and not damaged bacteria normalized to the total number of bacteria present in the images. Each reported percentage reported in Table 4 represents an average value referred to 4 different SEM scans to provide an estimate for the statistical relevance of the interaction effect of MoS_2_ NSs with bacteria.

Antibacterial, cytotoxicity and antiviral tests were analyzed using GraphPad Prism version 5 (GraphPad Software, Sand Diego, CA, United States). CC50 and EC50 were calculated from a sigmoidal dose-response curve. *P*-values of <0.05 were considered to be statistically significant.

### Fabrication Details

The starting commercialized bulk MoS_2_ powder (Sigma Aldrich, 69860, particle size 6 μm, density 5.06 g *mL*-1 at 25°C), graphite powder (Aldrich, 332461, mesh number of grains +100, >75%, particle size ∼300 mm) was exfoliated in de-ionized/elix water and Cyrene respectively, as a pure solvent using a tip sonicator (Bandelin Ultrasound SONOPLUS HD3200, maximum power 200 W), working frequency 20 kHz using KE-76 (a tapered tip with 6mm diameter) and MS-72 (a micro tip with 2 mm diameter). Whereas, for GO starting precursor graphite oxide (XIAMEN TOB NEW ENERGY TECHNOLOGY Co., TOB-2430, particle size 5–50 μm, density 0.3–0.4 g mL^–1^ at 25°C) was exfoliated using the same sonicator device as above using KE-76 micro tip at running amplitude of 70%.

### Exfoliation of GO NSs

Exfoliation of graphene oxide was carried out using 1600 mg of graphite oxide in 200 mL of de-ionized water using the same probe sonicator with KE-76 tapered tip at running amplitude of 70%. Net exfoliation energy of 194.4 kJ was obtained after exfoliating graphite oxide for 30 min with a substantial amount of stability in the given solvent. The procedure adopted for graphite oxide was different from MoS_2_ NSs.

### Controlled Centrifugation

The fundamental disadvantage of LPE of 2DMs is that it gives poly disperse nature of exfoliated 2D NSs in the given medium. These consist of un-exfoliated material, unstable dispersed NSs and of course separated and exfoliated nanoflakes in the same medium which could be a surfactant or a suitable solvent with matching surface tension that of the material. The larger dense particles tend to sediment faster than the less dense because of the earth’s gravitational field. In general, LPE produces a wide distribution of thickness and lateral sizes of 2D nanoflakes which makes it more preferable candidate to study its potential for fundamental applications of different areas of interest. Then, it is very crucial to separate this poly-disperse 2D NSs on the basis of their lateral size and thicknesses, what was achieved by means of a versatile bench top centrifuge model (Eppendorf Centrifuge 5810 R, Rotor F-34-6-38).

The un-exfoliated MoS_2_ NSs were removed by discarding the sediment at low centrifugal force of 100 g for 90 min. The obtained supernatant contained lower monolayer content with wide distribution of thickness and lateral sizes of NSs. The supernatant was then centrifuged at higher centrifugal force of 1000 *g* for 90 min followed by the re-dispersion of sediment into the fresh elix water in 3–5 mL. The above procedure was repeated twice with 2000 and 3000 *g* centrifugal force, respectively. Subsequently, the obtained supernatants were analyzed for further characterizations such as UV-Visible spectroscopy, Raman spectroscopy, ζ- potential, SEM and TEM.

The GO NSs dispersion was centrifuged at 3500 rpm for 30 min followed by the re-dispersion of the sediment in a smaller volume and separation of supernatant for further characterization analysis.

### UV-Visible Spectrum

Optical extinction spectra were acquired on Jasco V-530 UV-Vis spectrophotometer using 1 cm optics quartz cuvettes. The extinction spectra of MoS_2_ dispersion was analyzed to determine the Mean layer number < *N* >, Mean lateral size < *L* >, and Mean concentration < *C* > of the NSs by metrics as explained by Backes et al. (2014).

### ζ-Potential Measurements

Electrostatic stabilization is an important parameter to analyze the stability of the liquid exfoliated dispersions. The surface charges generated during the exfoliation can be attributed to electrophoretic mobility measurements (μ). So, these μ measurements were carried out on laser interferometric technique (Malvern Zetasizer Nano system) with irradiation from 633 nm He-Ne laser. The samples were injected in folded capillary cells, and the μ value was measured using a combination of electrophoresis and laser Doppler velocimetry techniques. The Henry’s equation (see Supplementary Material) was used to estimate the ζ-potential from the μ data. For the possible upper and lower limits of the ζ-potential, Henry’s equation was approximated to both the Huckel and Smoluchowsky limits (see Supplementary Material). The reason for this approximation is due to the particular solvent-sample relationship; Henry’s equation is approximated to get an estimate of surface charge values of exfoliated NSs. All the measurements were carried out at 25°C.

### Raman Spectroscopy

A confocal Raman microscope (Jasco, NRS-3100) was used to obtain Raman and photoluminescence spectra. The 514 nm line of an air-cooled Ar+ laser (Melles Griot, 35 LAP431 220), was injected into an integrated Olympus microscope and focused to a spot diameter of approximately 3 μm by a 20x objective with a final 4 mW power at the sample. A holographic notch filter was used to reject the excitation laser line. The Raman backscattering was collected using a 0.1 mm slit and a diffraction lattice of 1200 grooves/mm, corresponding to an average spectral resolution of 8 cm^–1^. Solutions were left evaporating on Si substrates, and it took 60 s to collect a complete data set by a Peltier-cooled 1024 × 128 pixel CCD photon detector (Andor DU401BVI). Raman micro-spectroscopic measurements of both GO and MoS_2_ samples were at least triplicated for scope of reproducibility and as a check of spatial homogeneity of the samples. Wavelength calibration was performed by using cyclohexane as a standard.

### SEM for Morphological Analysis

Morphological analyses of samples were performed with a scanning electron microscope (SEM) JEOL-JSM 5310 (CISAG laboratory, at University of Naples, Federico II). The SEM operating at 15 kV, is equipped with energy dispersive X-Ray spectroscopy (EDS); data were processed with INCA version 4.08^1^. The samples were metalized with gold by using a sputter coater. Oxford Instruments (2006): INCA - The microanalysis suite issue 17a + SP1 – Version 4.08. Oxford Instr. Anal. Ltd., Oxfordshire, United Kingdom.

### TEM for Material Morphological Analysis

TEM micrographs were collected using a FEI TECNAI G2 S-twin 200kV apparatus operating at 120 kV (LaB6 source). A drop (5 μL) of flakes (suspension in water) was transferred on carbon-coated copper grids and then left at room temperature until the solvent was completely evaporated. Concerning samples containing both viruses and nanomaterials, a drop (5 μL) of suspension was deposited onto a formvar/carbon TEM grid until the solvent was completely evaporated and then the sample was stained with phosphotungstic acid (2%, pH 6.5) for 30s to enhance the contrast.

### Dynamic Condition to Measure Antibacterial Activity of MoS_2_ NSs

The antibacterial activity was then further characterized by performing the plate microdilution method, according to the guidelines established by the National Committee on Clinical Laboratory Standards (NCCLS).

To uniform the bacterial suspension for this antimicrobial assay, fresh colonies of each strain, grown on agarized Brain Heart Infusion (brain infusion solids 12.5 g/L, beef heart infusion solids 5 g/L, proteose peptone 10 g/L, sodium chloride 5 g/L, glucose 2 g/L and disodium phosphate 2.5 g/L), were inoculated in Brain Heart Infusion liquid and incubated at 37° C overnight. The bacteria cells were harvested via centrifugation (4000 rpm for 10 min). They were washed three times with deionized water to remove residual growth medium constituents. The pellets were then suspended in ultrapure water, PBS buffer and Brain Heart Infusion liquid broth. Bacterial cell suspensions were diluted in the specific buffers or solutions to obtain cell samples having a turbidity (*OD*600) corresponding to 1 × 10^8^ CFU/mL. Hundred and eighty μl of cells in different buffers were incubated with 20 μl of fresh nanosheet suspensions (12.5–1.56 μg/ml) for 3–6–20 h in Brain Heart Infusion broth with shaking at 37°C. The viability was evaluated by measuring *OD*600. The tests were performed in triplicate.

### Antibacterial Assay of GO NSs

Susceptibility testing was performed following the broth microdilution method outlined by the National Committee on Clinical Laboratory Standards (NCCLS) using sterile 96-well microliter plates. One bacterial species for each group, based on the Gram classification, were selected: for Gram-negative bacteria, we selected (*E. coli* ATCC CRM-11229); for Gram-positive bacteria, we selected (*S. aureus* ATCC 6538). The dilutions (25 to 0.39 μg/mL and 100 to 0.39 μg/mL) of each NSs (GO-1 = 1400 μg/mL and GO-2 = 600 μg/mL) were prepared in water at a volume of 100 μL/well.

Each well was inoculated with 50 μl of the standardized bacterial inoculum, corresponding to a final test concentration of approximately 5 × 10^5^ CFU/mL. The microbial growth was observed after 3, 6, and 20 h of incubation at 37°C. The positive controls consisted of ampicillin and vancomycin for *E. coli* and *S. aureus*, respectively. The bacterial inhibition percentage (IC%) was determined according to the following equation:

(19) $$I\,C\%=\left[1-\frac{\begin{array}{c} (OD600ofthetestsample-\\ OD600oftheblank) \end{array}}{\begin{array}{c} (OD600ofthenegativecontrol-\\ OD600oftheblank) \end{array}}\right]\times 100$$

where *OD*600 is the optical density of the sample measured at 600 nm. The tests were performed in triplicate.

### Viral Strains and Cell Culture Conditions

HSV-1 (strain SC16), HSV-2 (strain 333) and HSV-1-GFP were propagated on Vero cells monolayers. Vero cells (ATCC CCL-81) are African green monkey kidney cells and they were grown in MEM supplemented with 10% fetal bovine serum (FBS). Measles virus (strain Edmonston) was propagated on Vero-hSLAM cells (ECACC 04091501). Vero/hSLAM are Vero cells transfected with a vector plasmid (pCXN2) and an expression plasmid (pCAG-hSLAM) coding for the human measles virus’s receptor, the signaling lymphocytic activation molecule (SLAM). Vero-hSLAM cells were grown in MEM supplemented with 10% FBS and selected in 0.4mg/ml Geneticin (G418).

### Determination of Viability (MTT Assay)

The viability of Vero cells was determined through MTT assay (Sigma Aldrich, code 11465007001). 2 × 10^4^ cells/well were treated with different concentrations ranging from 0.39 to 100 μg/mL for a total of 24 h in 96 wells plate. The day after MTT powder was added for 3 h and then the purple formazan crystals were dissolved in dimethyl sulfoxide (DMSO) (100 μL/well). Finally the absorbance was recorded on a microplate reader at a wavelength of 570nm. Not treated cells represented the positive control (CTR+), meanwhile cells treated with DMSO were the negative control (CTR−). The tests were performed in triplicate.

### Co-treatment With MoS_2_ and GO NSs

To evaluate the effect of the NSs on HSV-1 infectivity inhibition, a co-treatment experiment was performed: the cells were incubated with different concentrations of MoS_2_ (0.39, 0.78, 1.5, 3.125, 6.25, 12.5, 25, and 50 μg/mL) and GO NSs (10, 25, 50, and 100 μg/mL) in the presence of the virus (10^3^PFU/cell) for 1 h at 37°C. At the end of the treatment, the cell monolayer was washed with Phosphate Buffer Saline (PBS) 1X and incubated for 48 h in MEM supplemented with carboxymethylcellulose. After 2 days, the cells were fixed and stained with 0.5% crystal-violet, and the plaques were counted. The percentage of viral inhibition was calculated compared to the untreated HSV-1 control (CTR-) as followed:-

(20) $$\%\,v\,i\,r\,a\,l\,i\,n\,h\,i\,b\,i\,t\,i\,o\,n=\left[100-\frac{\begin{array}{c} (plaquescountedinthe\\ testsample) \end{array}}{\begin{array}{c} (plaquescountedinthe\\ negativecontrol) \end{array}}\right]\times 100$$

Not infected and not treated cells represented the positive control (CTR+), meanwhile infected cells were the negative control (CTR−). The tests were performed in triplicate.

### Virus Pre-treatment With MoS_2_ and GO NSs

To evaluate the effect of the MoS_2_ NSs on HSV-1 infectivity inhibition, a virus pre-treatment experiment was performed. MoS_2_ and GO NSs were added to the virus (1 × 10^4^ PFU/cell) and incubated for 1 h at 37°C at different concentrations (10, 25, 50, 75, and 100 μg/mL). After incubation, each mixture (virus + NSs) was diluted and the virus was infected with MOI of 0.01 PFU/cell. The dilutions were added to Vero cells monolayers for 1 h. At the end of the treatment, the cell monolayer was washed with PBS 1X and incubated for 48 h in MEM supplemented with carboxymethylcellulose. After 2 days, the cells were fixed and stained with 0.5% crystal-violet, and the plaques were counted. The experiments were performed in triplicate.

### Cell Pre-treatment With MoS_2_ and GO NSs

The NSs, used at the same concentrations as the other assays, and the positive control dextran sulfate (PMID: 10618073) (Sigma: 67578) were added on Vero cells and incubated for 1 h at 37°C. The virus was added to a MOI of 0.01 PFU/cell for 1 h at 37°C. Finally, the cells were incubated with CMC for 48 h at 37°C. Cells were fixed, stained and the number of plaques was scored.

### Post-treatment With MoS_2_ and GO NSs

Vero cells were incubated with HSV-1 (MOI 0.01 PFU/cell) for 1 h at 37°C, after that the virus was removed and the NSs were added at the same concentrations as the other assays and incubated for 1 h at 37°C. Acyclovir (Sigma: A7678) was used as positive control. Then the cells were incubated with CMC for 48 h at 37°C. The cells were then fixed, stained and the plaques counted.

### Safety Issues

The bacterial and viral strains used in this study belong to biosafety level 2, based on *U.S. Public Health Service Guidelines*. Therefore, we adhered to standard laboratory safety procedures, which included disinfection of laboratory surfaces before and after completing the experiments and the use of adequate personal protective equipment.

## Data Availability Statement

All datasets generated for this study are included in the article/Supplementary Material.

## Author Contributions

MS conceived the experiments, fabricated and characterized the nanomaterials, analyzed the results, and wrote the manuscript. CZ contributed to the viral experimental organization, data production, data analysis, text composition, analyzed the results, and wrote the manuscript. LA conceived the experiments and analyzed the results. FB analyzed the results and wrote the manuscript. AC contributed to the viral experimental, data production, and data analysis. AD fabricated the GO NSs. MD contributed to the bacteria-nanomaterial modeling. RD and AV analyzed the samples by TEM microscopy. VF contributed to the bacterial experimental organization, data production, data analysis, and text composition. GF contributed to the experimental supervision, experimental organization, data analysis, and text composition. CI contributed to the data analysis and text composition. MR analyzed the samples by SEM microscopy. MoV contributed to the data analysis, data production, bacteria-nanomaterial modeling, and text composition. MiV contributed to the data analysis and text composition. MG contributed to the experimental conception, data analysis, text composition, and main supervision. CA conceived the experiments, data analysis, bacteria-nanomaterial modeling, text composition, and main supervision. All the authors contributed to the article and approved the submitted version.

## Conflict of Interest

The authors declare that the research was conducted in the absence of any commercial or financial relationships that could be construed as a potential conflict of interest.

## Footnotes

1 https://www.brandonu.ca/microscope/files/2010/08/pdf_3.pdf
