# Supplementary material for: Combating Actions of Green 2D-Materials on Gram Positive and Negative Bacteria and Enveloped Viruses
Source: Front Bioeng Biotechnol. 2020 Sep 28;8:569967. doi: 10.3389/fbioe.2020.569967 (PMC7549698; doi:10.3389/fbioe.2020.569967)
Supplement: Supplementary file 1 [file Data_Sheet_1.pdf]

frontiers

Supplementary Material

Combating actions of green 2D-materials on gram positive and negative bacteria and enveloped viruses

Manjot Singh[#1], Carla Zannella[#2], Veronica Folliero[2], Rocco Di Girolamo[3], Francesco Bajardi[1,4], Annalisa Chianese[2], Lucia Altucci[5], Achille Damasco[1], Maria Rosaria Del Sorbo[6], Concetta Imperatore[7], Manuela Rossi[9], Mohammadhassan Valadan[1], Michela Varra[7], Alessandro Vergara[3], Gianluigi Franci[9], Massimiliano Galdiero[2*] and Carlo Altucci[1,4*]

[1] Laboratory of Bio-Nano-Photonics, Department of Physics “Ettore Pancini”, University of Naples “Federico II”, via Cintia 21, 80126, Naples, Italy

[2] Department of Experimental Medicine, University of Campania “Luigi Vanvitelli, via Santa Maria di Costantinopoli 16, 80138, Naples, Italy

[3] Department of Chemical Sciences, University of Naples “Federico II”, via Cintia 21, 80126 Naples, Italy

[4] Istituto Nazionale di Fisica Nucleare (INFN) Sez. di Napoli, via Cintia 21, 80126, Naples, Italy

[5] Department of Precision Medicine, University of Campania “Luigi Vanvitelli”, via Santa Maria di Costantinopoli 16, 80138, Naples, Italy

[6] Istituto Statale d’Istruzione Superiore “Leonardo da Vinci”, via F. Turati Poggiomarino, Naples, Italy

[7] Department of Pharmacy, University of Naples “Federico II”, via D. Montesano 9, 80131 Naples, Italy

[8] Department of Earth, Environmental and Resources Sciences, University of Naples “Federico II”, via Cintia 21, 80126, Naples, Italy

[9] Department of Medicine, Surgery and Dentistry "Scuola Medica Salernitana", University of Salerno, 84081 Baronissi SA, Italy

[#] These authors have contributed equally.

*** Correspondence:**
Carlo Altucci
caltucci@unina.it
Massimiliano Galdiero
massimiliano.galdiero@unicampania.it

Graphical Abstract

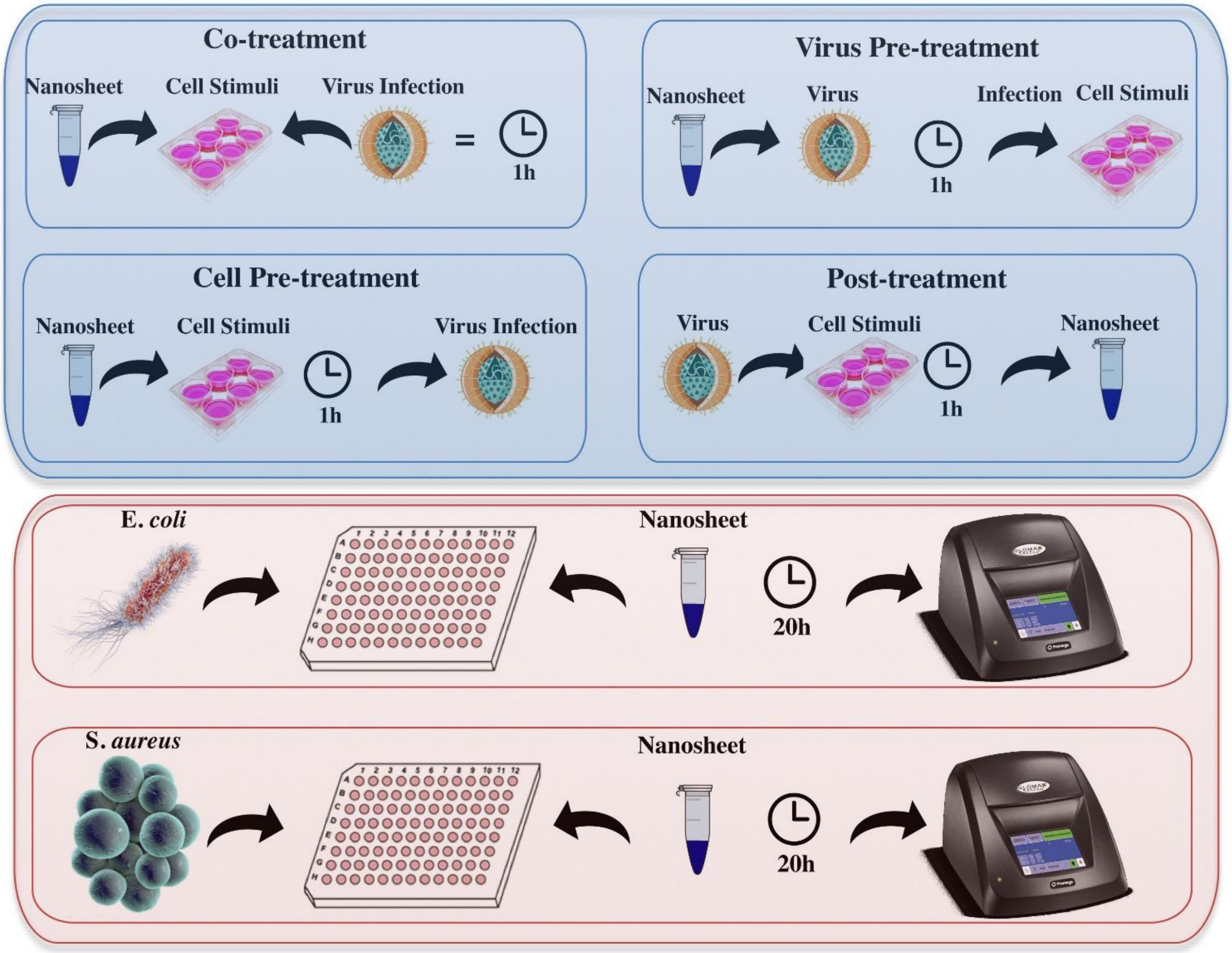

Exfoliation Parameters

Basically, in the first step of exfoliation, an initial concentration (C_i) of 5 mg/mL was taken (24 mg of bulk MoS_2 powder was dissolved in 12 mL of helix water by 5-10 minutes of vortex). Then, the solution was probe sonicated using KE-76 tapered tip for one hour at running amplitude of 45%.

Materials characterization

UV- Visible Spectroscopy

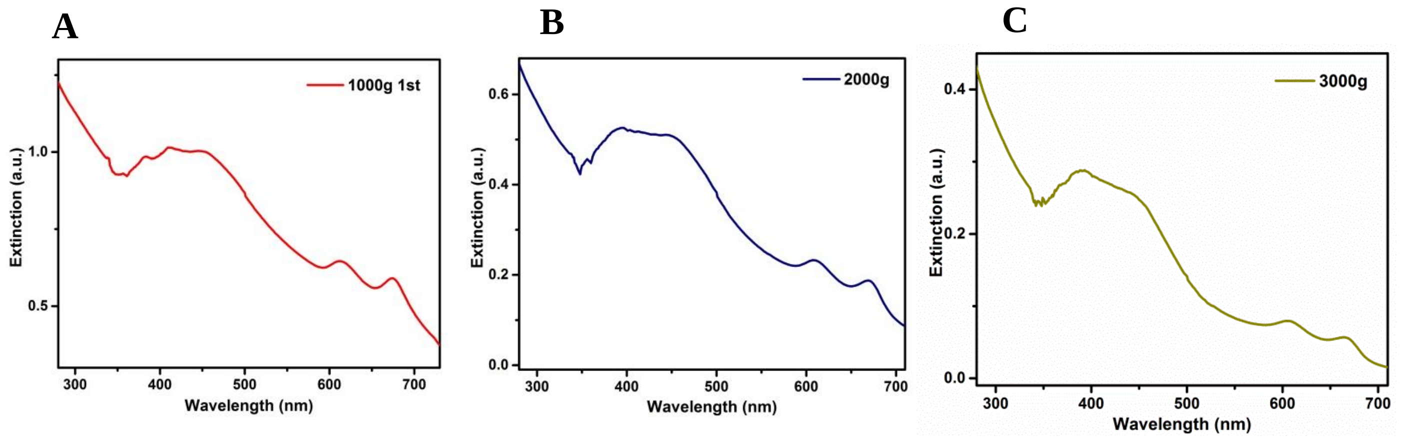

***Figure S1*. UV-Visible of MoS_2 NSs**. Panel (A-C): UV-visible extinction spectra of 2D MoS_2 NSs dispersion at C_i=5 mg/mL (volume of the solvent 12 mL) at 1000g, 2000g and 3000g showing the effect of increase in the centrifugal force results in reduction of A-exciton and B-exciton wavelength.

By reading the wavelength position of A-exciton, λ_A, and the extinction at the B-exciton maximum, Ext_B, and at 345 nm, Ext_{345}, one can estimate the average number of layers, N, and lateral size, L, through equations 1 and 2 as shown in (A-C)

$$N = 2.3 \, x \, 10^{36} e^{\frac{-54888}{\lambda_A}} = 2.3 \, x \, 10^{36} e^{-44.3 E_A} \quad (1)$$

$$L \, (\mu m) = \frac{3.5 Ext_B \, / \, Ext_{345} - 0.14}{11.5 - Ext_B \, / \, Ext_{345}} \quad (2)$$

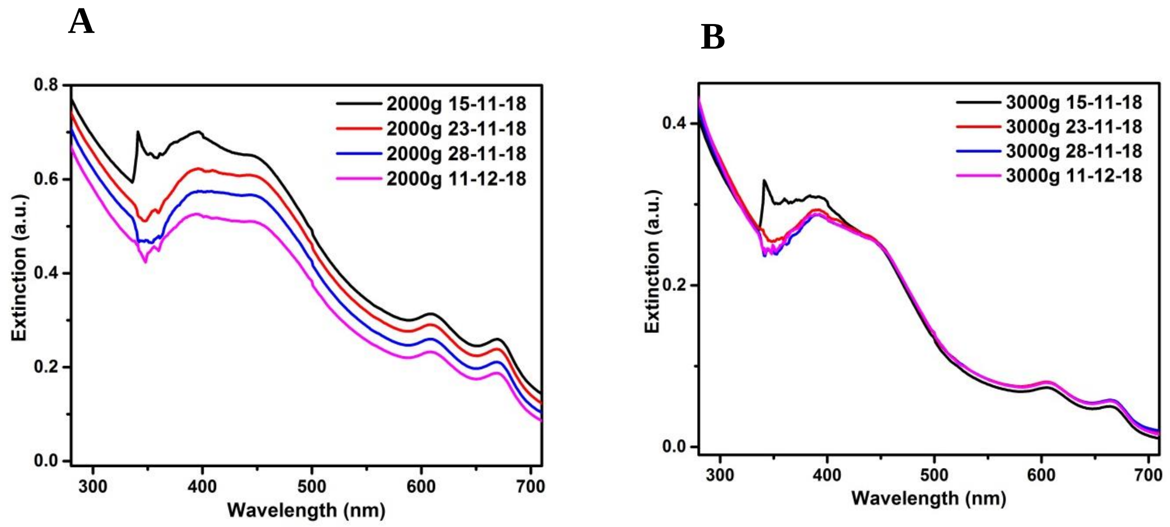

***Figure S2*. Stability measurements for the MoS_2 NSs.** (A-B) represents the extinction spectra of MoS_2 NSs at 2000g and 3000g measured for four weeks to analyze the stability of water exfoliated 2D NSs.

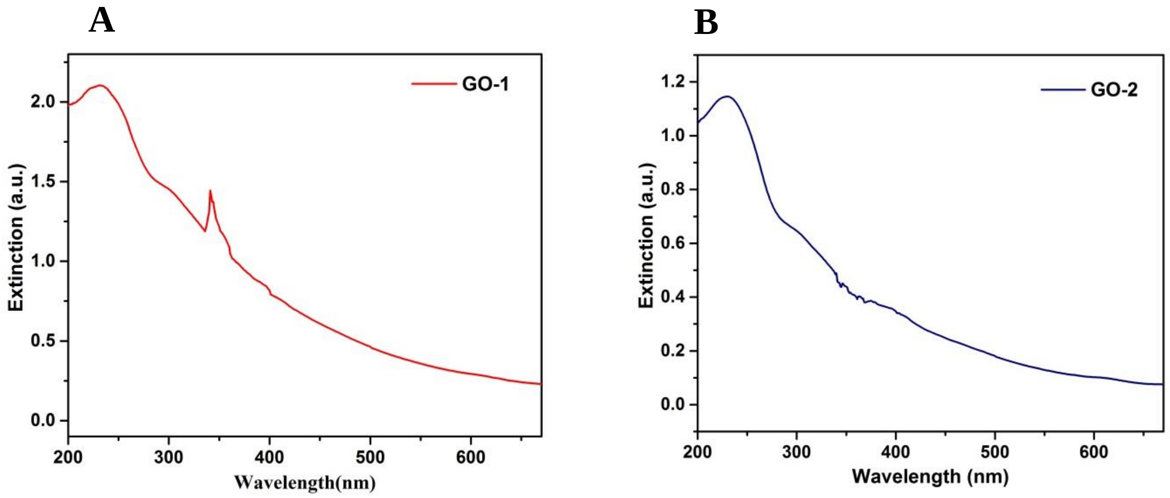

***Figure S3.* UV-Visible spectra of graphene oxide NSs.** (A-B) Extinction spectra of two different concentrations of GO, GO(1)= 1400 µg/mL and GO(2)= 600 µg/mL.

Raman spectroscopy analysis

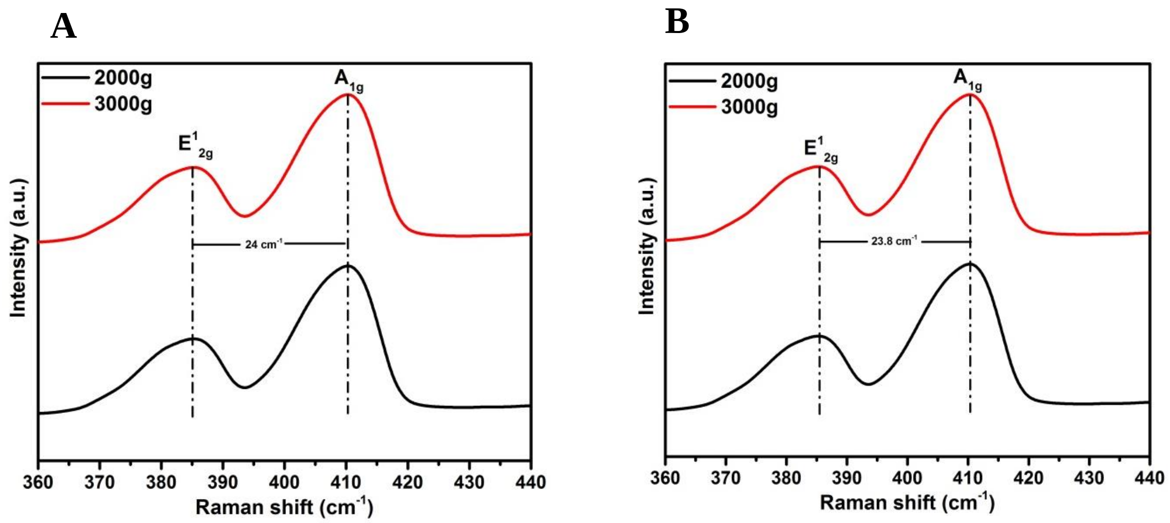

Figure S4. **Raman spectra of MoS_2 NSs.** Panel (A-B) Raman spectra of MoS_2 NSs at 2000g and 3000g represents the difference between the two main peaks of MoS_2 in the range of 23.8 cm^{-1} – 24 cm^{-1} which exhibits the number of layers from 2-4.

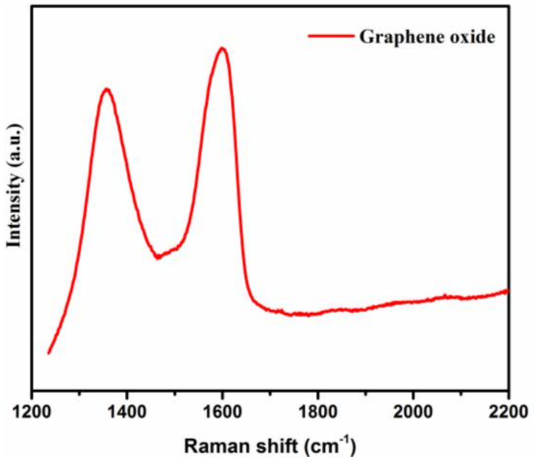

Figure S5. **Raman spectra of GO NSs.** Raman spectra of GO NSs at 1500g represents the two characteristic D and G bands of exfoliated GO NSs. The spectra shows the spatial homogeneity when deposited onto the substrate and recorded in different regions (Data not shown). The final concentration of GO NSs for this spectrum is 600µg/mL. The I(D)/I(G) value is 0.93, whereas I(G)/(2D) is 1.25

ζ- Potential of GO NSs

**Centrifugal force (g)**	**Zeta potential *(ζ) mV***
**2000g**	**-48.0 mV**
**3000g**	**-47.5 mV**
**3500g**	**-49.4 mV**

Table S1. ζ-potential values of GO NSs. ζ–potential values of GO NSs at three different centrifugal forces represents a high value corresponding to highly stable dispersion in pure water.

During ζ-potential measurements, an electrical field is applied across the sample, inducing the movement of charged particles. The ratio between the nanoparticles velocity and the external applied field, known as electrophoretic mobility (μ), is then measured and converted to the ζ-potential using the Henry's equation:

$$\mu = \frac{2\varepsilon z f(k\alpha)}{3\eta} \quad (3)$$

where, ε and η are the dielectric constant and the absolute zero-shear viscosity of the medium, respectively.

MTT assay

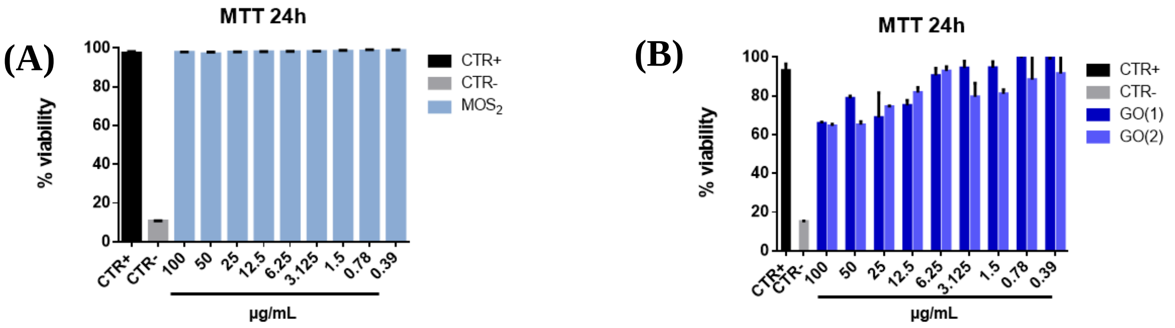

***Figure S5*:** (A) Cell viability of Vero cells pre-treated with MoS_2 at various concentrations. (B) Cell viability of Vero cells presented as percentage of control after 24 h exposure to different concentrations of GO in water (200-0.39 µg/ml). 1 and 2: same compounds with different concentrations (1=1400 µg/mL; 2=600 µg/mL).

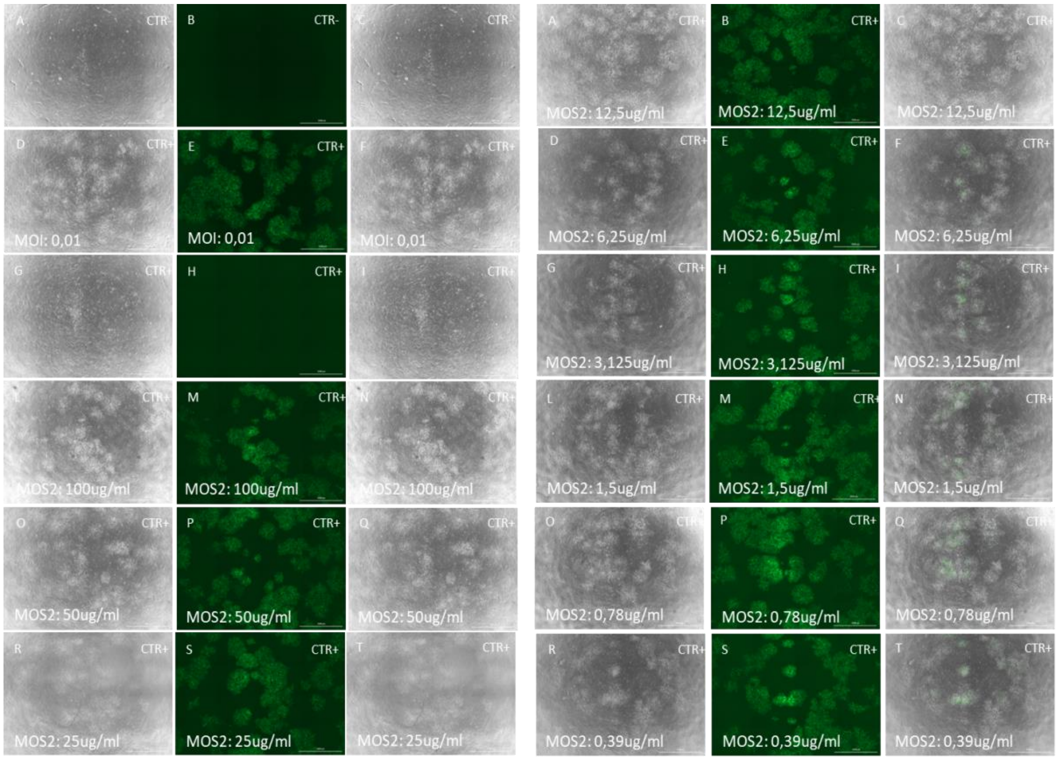

Figure S6. GFP expression in Vero cells infected by HSV-1 at 0.01 multiplicities of infection (MOIs) at 48 h post infection. The fluorescence amount in the control virus and that measured for various concentrations of MoS_2 nanoflakes is the same, confirming the absence of antiviral effect.
